# From biogenesis to deep modeling: a holistic review of miRNA–disease prediction computational methods with experimental comparison

**DOI:** 10.1093/bib/bbaf736

**Published:** 2026-01-19

**Authors:** Siya Xie, K L Eddie Law

**Affiliations:** Faculty of Applied Sciences, Macao Polytechnic University, Macao 999078, SAR, China; Faculty of Applied Sciences, Macao Polytechnic University, Macao 999078, SAR, China

**Keywords:** computational methods, microRNA, miRNA–disease association, MDA prediction

## Abstract

Abnormal dysregulation of microRNAs (miRNAs) expression may lead to a wide spectrum of diseases, and as miRNAs play pivotal roles in disease pathogenesis, diagnosis, and therapy, identifying potential miRNA–disease associations (MDAs) is essential for discovering new diagnostic biomarkers, developing targeted therapeutic strategies, and advancing personalized medicine. Traditional wet-lab experiments are time-consuming, expensive, and consume a lot of resources. Hence, various computational approaches should be developed as auxiliary *a priori* tools. In the following text, we compile different methods proposed for MDA prediction over the past decade. First, we analyze the data resources supporting MDA studies and introduce approaches for quantifying similarities among MDAs. Second, we comprehensively review 66 computational methods, classify them into five categories, and present comparative experimental analyses on selected methods to identify future research directions. To enhance accessibility, we upload a summary of discussed methods to a GitHub repository (https://github.com/xiesiya/miRNA-disease-association-methods). This review provides comprehensive background knowledge on computational methods for future MDA prediction research.

## Introduction

Genetic information is transmitted from DNA to messenger RNA (mRNA) through a process termed *transcription*. Subsequently, within the ribosome-transfer RNA cofactor complex, mRNA is translated in accordance with the genetic instructions encoded in DNA, thereby facilitating protein synthesis. Beyond protein-coding mRNAs, cells harbor a vast array of functional noncoding RNAs (ncRNAs) that do not encode proteins [1]. Within the broader ncRNA hierarchy, micro RNA (miRNAs) interact with long noncoding RNA (lncRNA), circular RNA (circRNA), and small interfering RNA (siRNA), etc., forming a regulatory networkstructures, as outlined in Supplementary Text 1. These molecules are directly involved in the regulation of gene expression. Among them, miRNAs have attracted particular attention because of their ubiquity and essential role in posttranscriptional gene regulation [2].

**Figure 1:**
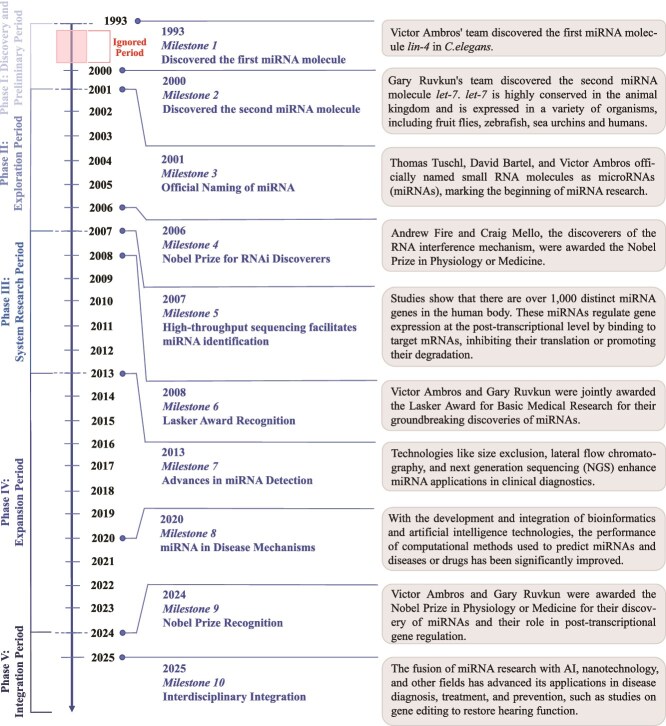
The history of miRNA research: key developments and advancements in miRNA studies, breakthroughs, and emerging directions.

MiRNAs are ncRNAs of 20–25 nucleotides (nt) in length that fine-tune gene expression at the posttranscriptional level through complementary base-pairing with target mRNAs, thereby repressing translation or promoting mRNA degradation [3, 4]. The maturation process of miRNAs can be summarized as follows: primary miRNA (Pri-miRNA) is processed into precursor miRNA (Pre-miRNA) by Dicer enzyme in the cell nucleus, cleaved by Dicer enzyme in the cytoplasm, and finally integrated into the miRNA-containing RNA-induced silencing complex (miRISC), which mediates the degradation or translation inhibition of target mRNA through base pairing (see Supplementary Text 2 for details). Ubiquitously found in plants, animals, and even some viruses, miRNAs are integral to nearly every critical biological process, including cell proliferation, differentiation, development, and apoptosis [5–7]. To date, >57 000 human miRNAs have been discovered [8], which collectively regulate the expression of thousands of genes at the posttranscriptional and translational levels [9, 10]. Over the past three decades, miRNA research has seen exponential growth and gained widespread attention. To systematically overview this evolving field, we divide it into five distinct developmental phases. The detailed descriptions of these phases provided in Supplementary Text 3. Figure 1 shows the key milestones in miRNA research from 1993 to 2025.

Unfortunately, dysregulation of aberrant miRNA expression may lead to a variety of diseases, including cancer [11, 12], cardiovascular disease [13], and neurodegenerative diseases [14]. For instance, studies find that *miR-214* is highly expressed in the sera of elderly patients with acute myocardial infarction, and may inhibit cardiomyocyte apoptosis by repressing the expression of its target genes, including *PUMA*, *PTEN*, *Bax*, and *Caspase-7* [15]. Additionally, *miR-5p* exhibits elevated expression in clinical serum samples from patients with psoriasis [16]. In patients with spinal cord glioma, *miR-106a-5p* is significantly upregulated. Its overexpression in spinal cord glioma tissues may influence cell proliferation, migration, invasion, and apoptosis by targeting *CELF-2*, thereby providing a potential strategy for future clinical treatment of spinal cord glioma [17]. Furthermore, *hsa-miR-503* and *hsa-miR-96* play critical roles in the immune function, pathogenesis, and prognosis of squamous cell lung carcinoma, serving as key determinants of the disease’s progression, immune landscape, and clinical outcome [18]. Similarly, *miR-34a* has been identified as a potential anti-cancer miRNA for gene therapy targeting bone metastatic cancer [19]. These studies have shown that miRNAs play a key role in disease progression, diagnosis, and treatment. Therefore, identifying potential MDAs is crucial for discovering new diagnostic biomarkers, developing targeted therapeutic strategies, and advancing personalized medicine.

Although traditional wet-lab experiments can accurately validate MDAs, they are usually expensive and time-consuming. Computational methods may effectively reduce experimental time, save cost and resources, and therefore are good preliminary validation tools. As early as 2010, Jiang *et al.* [20] proposed a network-based computational method that constructs a *human phenome-miRNAome network* to prioritize disease-associated miRNAs and thereby infer potential MDAs. In addition, Luo *et al.* [21] proposed the BRWH (using unbalanced bi-random walk on heterogeneous network) model. This model utilizes unbalanced bi-random walk to propagate the association between known miRNAs and diseases to unknown miRNAs and diseases, thereby predicting potential MDA. This model achieved good prediction results. However, network-based computational methods are still difficult to automatically learn nonlinear high-order relationships. Recently, a computational method based on matrix factorization, the SMAP model [22], was proposed. This method integrates similarity information into an optimized matrix factorization framework to predict potential MDA. Although previous matrix factorization-based computational methods have been successful in predicting MDAs, they have the problem of being sensitive to neighborhood information and highly dependent on the input data source. Subsequently, machine learning methods also showed good performance. Chen *et al.* [23] proposed a regularized least squares (RLS)-based method employing a semi-supervised learning algorithm to predict MDAs. The gradient boosting tree algorithm shows excellent advantages in processing large-scale data sets and improving prediction accuracy. For example, the KS-CMI model [24] combines the denoising autoencoder and the categorical boosting algorithm (CatBoost) to enhance feature representation and enhance prediction robustness through balance theory. However, with the rapid growth of relevant data in recent years, traditional machine learning algorithms cannot adapt to complex and variable data and cannot automatically mine high-order features.

With the rapid deep learning development, the advantages in capturing nonlinear associations within MDAs are crucial. Liu *et al.* [25] proposed SMALF, which employed stacked autoencoders (SAEs) to learn latent representations of miRNAs and diseases. The design integrated multiple similarity-based features into comprehensive feature vectors, and utilized the extreme gradient boosting (XGBoost) algorithm for final MDA prediction. However, computational methods based on non-graph deep learning cannot handle graph structure data and ignore the correlation between samples. Lately, since MDAs are mostly and essentially graph-structured data, some methods were developed based on graph convolutional networks (GCNs) to work in this field and achieve noticeable results. For example, Tang *et al.* [26] proposed MMGCN model, which employs a GCN encoder to acquire features of miRNAs and diseases under different similarity views. Furthermore, this model uses a multichannel attention mechanism to adaptively learn the importance of different features, thereby enhancing the latent representation for association prediction. To obtain miRNA and disease embeddings with better generalization ability, Sheng *et al.* [27] employed graph contrastive learning to perform multitask prediction of MDAs, lncRNA–disease associations, and lncRNA–miRNA interactions. Although computational methods based on graph neural networks (GNNs) have achieved good predictive performance in recent years, the black-box nature of this method has brought new challenges to biological interpretability.

**Figure 2:**
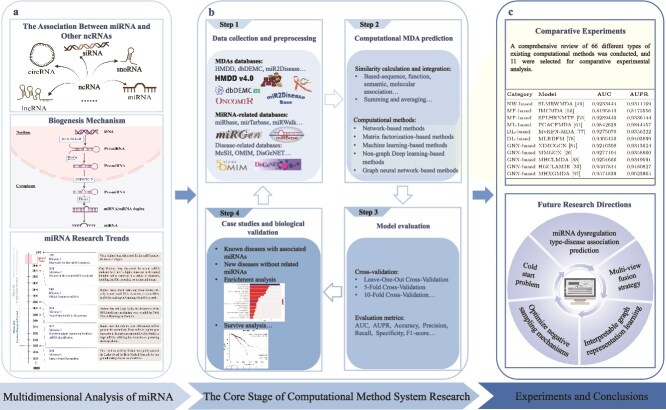
Overall framework of the review.

In summary, numerous computational methods have been applied to uncover potential MDAs. In the following text, we classify different MDA identification approaches into five categories: stochastic network-based methods (NW-based), matrix factorization-based methods (MF-based), machine learning-based methods (ML-based), non-graph deep learning-based methods (DL-based), and GNN-based methods. Although GNNs are a branch of deep learning, in recent years, GNN-based methods have demonstrated superior performance in aggregating node information compared with traditional non-graph deep learning approaches. This is primarily because MDAs inherently exhibit graph-structured characteristics. GNN-based methods can operate directly on the heterogeneous MDA graph. Therefore, we categorize these methods separately to enable a clearer discussion and summary. The overall framework of this review is demonstrated in Fig. 2: (i) the biological mechanism of miRNA and its relationship with other ncRNAs; the historical development of miRNA research in detail. (ii) A systematic research framework for computational methods to predict MDAs: (a) multi-source data integration; (b) using computational methods to predict MDAs and classifying computational methods into five categories; (c) cross-validation and evaluation; (d) biological validation. (iii) Comparative experiments and summaries of different categories are then presented.

## Datasets and similarity

### Public datasets

The rapid advancement of miRNA research has driven the development of dedicated databases that systematically curate experimentally validated MDAs. These databases serve as foundational resources for computational methods and biomedical applications. Table 1 provides a comprehensive summary of key public MDA databases, including their websites, miRNA/disease statistics, etc.

**Table 1:** Summary of MDA databases

Databases	Website	Species	miRNAs number	Diseases number	Associations	Authors and year
HMDD v4.0 [28]	http://www.cuilab.cn/hmdd	Human	1817	2360	53 357	Cui *et al*. 2007, updated in 2023
dbDEMC 3.0 [8]	https://www.biosino.org/dbDEMC/index	Human	2584	149	160 799	Yang *et al*. 2010, updated in 2022
miRCancer [29]	http://mircancer.ecu.edu/	Human	57 984	196	9080	Xie *et al*. 2013, updated in 2020
miR2Disease [30]	http://www.mir2disease.org/	Human	349	163	3273	Jiang *et al*. 2008

These databases scale ranges from miR2Disease’s curated 3273 MDAs to dbDEMC’s expansive 160 799 MDAs. Researchers can leverage these differences to select databases aligned with specific objectives. By integrating data from these resources, computational methods can predict new MDAs through network analysis, machine learning, or deep learning methods.

### Similarity matrices

Disease similarity and miRNA similarity are calculated by extracting information from various specialized databases on miRNAs or diseases, which helps to quantify the association between diseases and the association between miRNAs. These similarity matrices serve dual purposes: representing intrinsic features of biological entities, and enabling computational methods to capture latent associations from heterogeneous data sources. Below we introduce key similarity computation paradigms by category.

#### Disease similarity matrices

**I. Disease semantic similarity (DSS)** leverage hierarchical disease ontologies for associations quantification. The ontology-based disease semantic similarity ($\mathrm{DSS}_{1}$) constructs directed acyclic graphs (DAGs) using Medical Subject Headings (MeSH) descriptors, where disease nodes are weighted by their information content in the ontology [26]. To address the limitation of uniform layer weighting in $\mathrm{DSS}_{1}$, an enhanced semantic similarity $\mathrm{DSS}_{2}$ was proposed, incorporating disease co-occurrence frequencies across DAGs [31]. The final disease semantic similarity is $\mathrm{DSS} = (\mathrm{DSS}_{1} + \mathrm{DSS}_{2})/2$. The formulas and calculation processes for $\mathrm{DSS}_{1}$ and $\mathrm{DSS}_{2}$ are detailed in Supplementary Text 4.

**II. Disease functional similarity (DFS)** uses a target-based computational approach similar to MFS [26]. It measures the similarity between diseases by leveraging disease–gene associations and the degree of overlap in disease-related gene sets. For details of the calculation process, please refer to Supplementary Text 4.

#### miRNA similarity matrices

**I. miRNA functional similarity (MFS)** matrix is based on the assumption that miRNAs associated with similar diseases have similar effects. There are three ways to calculate the MFS matrix: based on known MDAs [32], disease functional similarity [33], and miRNA-gene association [26, 34]. The resulting MFS matrix provides pairwise functional similarity scores for all miRNAs. The calculation process of MFS is described in Supplementary Text 4.

**II. miRNA sequence similarity (MSS)** is determined by calculating the similarity matrix of miRNA sequences using the Needleman–Wunsch algorithm [26, 35] and Smith Waterman algorithm [36, 37]. The specific calculation process is described in Supplementary Text 4. The calculation can be performed using the pairwiseAlignment function in R package *Biostrings*, and the Needleman–Wunsch algorithm or Smith Waterman algorithm can be selected by adjusting the parameters “type=global” or “local.” To ensure global consistency, the raw scores are normalized based on the minimum and maximum values in the matrix. This normalized similarity provides a sequential representation of miRNA similarities across the dataset.

#### Gaussian interaction profile kernel similarity

In previous studies [38], to characterize the potential associations between miRNAs and diseases, it is commonly assumed that miRNAs with similar functions are more likely to be involved in diseases with similar phenotypes. Based on this assumption, researchers utilize known MDA data to construct binary interaction profiles for both miRNAs and diseases, and then calculate the Gaussian Interaction Profile Kernel (GIPK) similarity. Specifically, a binary vector is first generated for each miRNA to indicate its known associations with all diseases. Subsequently, the similarity between any two miRNA vectors is measured using a Gaussian kernel function, where the kernel bandwidth parameter is determined based on the total number of miRNAs and is set to 1 following previous work [38]. Similarly, the GIPK similarity for diseases is computed using the same approach. The detailed computational formulas for GIPK similarity are provided in Supplementary Text 4.

## Computational methods for predicting miRNA–disease associations

**Figure 3:**
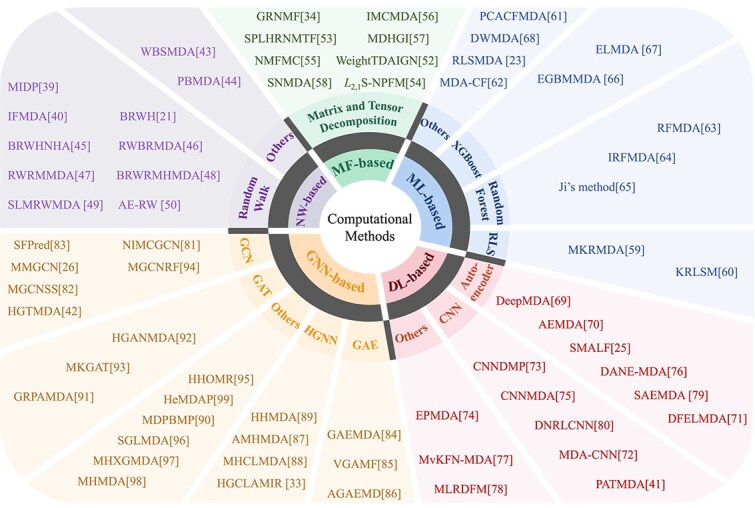
Classification of computational methods for MDA prediction. RLS, CNN, GCN, GAT, GAE, and Hypergraph Neural Network.

The increase in MDA datasets has promoted the development of various computational methods to uncover hidden patterns from complex biological networks. These methods aim to address challenges such as data sparsity, biological heterogeneity, and the need for interpretable predictions. Figure 3 provides a comprehensive classification overview of computational methods used for predicting MDAs. We focus on the computational methods published in the last decade. These methods are categorized according to their core technical contributions. For models that integrate deep learning with machine learning classifiers, we classify them based on the core innovation of their feature learning module. Overall, the methods can be systematically divided into five categories: NW-based, MF-based, ML-based, DL-based, and GNN-based method. Each category reflects distinct computational philosophies for feature extraction, association modeling, and prediction optimization.

### Network-based methods

In this section, we discuss traditional methods that predict potential MDAs based on network random walk strategies. The core idea of these approaches is to exploit network connectivity and topological structures to propagate probabilities from known associations across the network, thereby quantifying the relevance between unobserved nodes and known diseases or miRNAs.

The methodological evolution has progressed from early schemes that simply integrated miRNA and disease similarity networks [20], toward more sophisticated multi-network fusion frameworks that incorporate propagation algorithms such as random walk [39] and random walk with restart (RWR) [40]. In recent years, to mitigate the influence of network noise, studies such as [41, 42] have further introduced attention mechanisms to dynamically weight the importance of edges during the propagation process.

These methods share the advantage of iteratively aggregating information from neighboring nodes, effectively smoothing sparse association data and leveraging the topological properties of biological networks. Nevertheless, their performance remains highly dependent on the quality of similarity metrics and the completeness of the network structure, making them sensitive to sparsity and noise; moreover, they still struggle to automatically capture nonlinear and higher order relationships. In this section, we systematically reviewed 11 high-impact NW-based studies published in the last decade (see Table 2) and divided them into two categories.

**Table 2:** NW-based methods for predicting MDAs

Method	Description	Evaluation	Datasets	AUC values	Source Code
**MIDP**(Xuan et al., 2015) [39]	• Random walk; • Integration of disease similarity, miRNA similarity, and bilayer network topology	AUC values for 18 diseases	HMDD v2.0	0.862	Unavailable
**WBSMDA** (Chen et al., 2016) [43]	• Integrating within and between scores from heterogeneous biological datasets; • Applicable to diseases with unknown associated miRNAs	LOOCV	HMDD v2.0	0.8031	Unavailable
**IFMDA** (Liu et al., 2016) [40]	• RWR on a heterogeneous network; • Integrates DSS, DFS, miRNA-target gene, and miRNA-lncRNA associations	five-fold CV, LOOCV	HMDD v2.0	0.8476 (five-fold CV), 0.8049 (LOOCV)	Unavailable
**PBMDA** (You et al., 2017) [44]	• Path-based computational method; • Integrating MDA, MFS, DSS, and GIPK similarity; • Depth-first search	five-fold CV, LOOCV	HMDD v2.0	0.9172 (five-fold CV), 0.8341 (local LOOCV), 0.9169 (LOOCV)	Unavailable
**BRWH** (Luo et al., 2017) [21]	• Unbalanced bi-random walk; • Subgraph-based method for uncovering MDAs	five-fold CV	HMDD v2.0	0.8459	Unavailable
**BRWHNHA** (Yu et al., 2019) [45]	• Hybrid recommendation algorithm; • Unbalanced bi-random walk; • Integration of GIPK similarity	five-fold CV	HMDD v2.0	0.857	https://github.com/myl446/BRWHNHA
**RWBRMDA** (Niu et al., 2019) [46]	• RWR; • Binary logistic regression	LOOCV	HMDD v2.0	0.8076	Unavailable
**RWRMMDA** (Nguyen et al., 2021) [47]	• Improved RWR; • Integrates multiple similarities; • WKNKN pre-processing	five-fold CV, LOOCV	HMDD v2.0	0.9855 (five-fold CV), 0.9882 (LOOCV)	Unavailable
**BRWRMHMDA** (Qu et al., 2021) [48]	• RWR; • Multilayer heterogeneous networks	LOOCV	HMDD v2.0	0.8310	Unavailable
**SLMRWMDA** (Yao et al., 2024) [49]	• Sparse learning; • Multilayer random walks; • Heterogeneous network construction	five-fold CV, LOOCV	HMDD v2.0, HMDD v3.2	0.9335 (five-fold CV), 0.9368 (LOOCV)	https://github.com/HA1B1N/SLMRWMDA/tree/main
**AE-RW** (Lu et al., 2024) [50]	• Autoencoder; • Random walk; • miRNA-gene-disease heterogeneous network	five-fold CV	HMDD v3.2	0.9478	https://github.com/LUTGraphGroup/AE-RW_jiangjicheng

$^\dagger $ “Source Code” is derived from the corresponding paper. “Unavailable” indicates that the “Source Code” is not provided in the corresponding paper. “AUC” is the area under the receiver operating characteristic curve. LOOCV AUC values in the table default to global LOOCV.

#### Random walk

**I. miRNAs associated with diseases prediction (MIDP)** Xuan *et al.* [39] proposed MIDP, a random walk-based model for predicting disease-related miRNAs, particularly for diseases with partial known associations. Known disease-related miRNAs are labeled nodes, while others are unlabeled. Transition matrices $ M_{Q} $ (for labeled nodes) and $ M_{U} $ (for unlabeled nodes) are built, with higher weights $ r_{Q}> r_{U} $ assigned to labeled nodes to prioritize their influence. A random walker starts from labeled nodes, iteratively traverses the network based on functional similarity and local topology, and computes steady-state probabilities for unlabeled nodes as relevance scores. For diseases without known associations, MIDP utilizes a miRNA–disease two-layer network to combine miRNA and disease networks with inter-layer jump weights.

**II. Inferring MDAs by RW on a heterogeneous network (IFMDA)** Liu *et al.* [40] proposed IFMDA, addressing the limitations of single-dataset reliance and inadequate similarity measures in existing methods. The core innovation lies in constructing a heterogeneous network by integrating DSS, DFS, and MFS. The RWR algorithm is applied to this network to infer potential MDAs.

**III. Unbalanced bi-random walk-based algorithm on the heterogeneous network (BRWH)** Luo *et al.* [21] proposed BRWH, a method for predicting MDAs by integrating DSS and MFS into a heterogeneous network. The core innovation lies in the unbalanced bi-random walk algorithm, which separately performs RWs on the disease similarity network (DIN) and miRNA functional similarity (MIN) network to explore bipartite subgraph patterns. The algorithm iteratively extends miRNA and disease paths, controlled by parameters $ L_{1} $ and $ L_{2} $, to infer potential MDAs. The predicted relevance scores are computed as


(1) \begin{align*} & \mathrm{MIN:} \quad P_{t} = (1-\alpha) \cdot M \cdot P_{t-1} + \alpha \cdot A \end{align*}

(2) \begin{align*} & \mathrm{DIN:} \quad P_{t} = (1-\alpha) \cdot P_{t-1} \cdot D + \alpha \cdot A, \end{align*}

where $ t $ represents the iteration steps, $ M $ and $ D $ are transition matrices for MIN and DIN, respectively, and $ A $ is the known association matrix. $ \alpha $ ranging from 0 and 1 represents the decay factor.

**IV. Hybrid recommendation and unbalanced bi-random walk for MDA prediction (BRWHNHA)** Yu *et al.* [45] proposed the BRWHNHA method based on hybrid recommendation and unbalanced double random walk prediction MDA. The method integrates GIPK similarity into the MFS network and DSS network. It then calculates the transition probability matrix of the miRNA–disease bipartite network using a hybrid recommendation algorithm. Finally, unbalanced bi-random walk is performed on the heterogeneous network to infer potential MDAs.

**V. RW and binary regression-based MDA prediction (RWBRMDA)** Niu *et al.* [46] proposed RWBRMDA, a computational method integrating RWR and binary logistic regression to predict MDAs. The core methodology involves constructing an integrated miRNA similarity network based on MFS and GIPK similarity. For each miRNA, RWR is performed on the network to extract global association features, represented as stable probability vectors. These feature vectors, combined with known MDAs, are used as inputs for binary logistic regression to calculate association probabilities.

**VI. Improved RWR and integrating multiple similarities for MDA prediction (RWRMMDA)** Nguyen *et al.* [47] proposed the RWRMMDA method to solve the problems of data sparsity and incompleteness by integrating multiple similarity networks and the improved RWR algorithm. The method begins with a weighted K-nearest known neighbors (WKNKN) preprocessing step to impute missing associations, reducing the impact of data sparsity. DSS, MFS, and GIPK similarity are integrated to construct heterogeneous networks. An improved RWR algorithm is then applied, assigning transition probabilities based on node degrees in the heterogeneous networks, and the final prediction scores are derived from the weighted combination of disease-based and miRNA-based networks.

**VII. Biased RWR on multilayer heterogeneous networks for MDA prediction (BRWRMHMDA)** Qu *et al.* [48] proposed BRWRMHMDA, which integrates known MDAs, DSS, MFS, and GIPK similarity for miRNAs and diseases. The core innovation lies in the degree-based biased RWR mechanism, which adjusts transition probabilities based on node degrees to optimize the random walk process. The workflow involves constructing integrated similarity matrices for diseases and miRNAs, followed by executing BRWR on the multilayer network to iteratively update node probabilities until convergence, generating predicted association scores.

**Figure 4:**
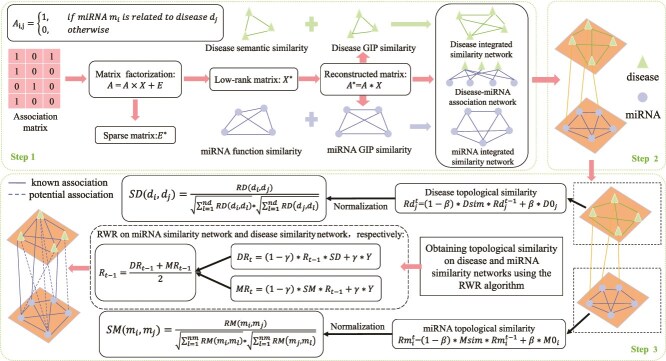
Flowchart of the SLMRWMDA, an MDA prediction model based on sparse learning and multilayer random walks. In the formula, $t$ denotes the iteration step, $\beta \in (0,1)$ is the random-walk damping factor, $D0_{j}$ and $M0_{i}$ are the normalized result. $\gamma \in (0,1)$ is the restart probability, and $R$ is the MDA score matrix.

**VIII. MDA prediction model based on sparse learning and multilayer RW (SLMRWMDA)** Yao *et al.* [49] proposed SLMRWMDA (see Fig. 4), which combines sparse learning and multi-layer RW algorithm to predict MDAs. The method first reconstructs the MDA matrix through sparse learning to remove noise and enrich association information, simultaneously generating the initial probability matrix for the RWR algorithm. A heterogeneous network is constructed using disease similarity, miRNA similarity, and the reconstructed association network. The multilayer RW algorithm captures topological features of diseases and miRNAs, computing probabilities to predict potential MDAs.

**IX. AutoEncoder and implementing RW on miRNA–gene–disease heterogeneous network (AE-RW)** Lu *et al.* [50] proposed AE-RW, to predict MDAs using the miRNA–gene–disease heterogeneous network. Feature extraction is performed using an autoencoder to capture high-order representations and a RW algorithm to explore network topology. These features are then concatenated and fed into a deep neural network (DNN) for MDA prediction.

#### Others

Chen *et al.* [43] proposed WBSMDA, a computational method for predicting MDAs, particularly targeting diseases or miRNAs without known links. The method integrates known MDAs, MFS, DSS, and GIPK similarity. The core innovation lies in the “within-score” and “between-score” mechanisms: “within-score” evaluates the similarity between the target miRNA and known disease-associated miRNAs, while “between-score” assesses the similarity between the target miRNA and nonassociated miRNAs. These scores are combined to generate the final association prediction score. You *et al.* [44] proposed PBMDA, a path-based computational method for MDA prediction. The core innovation lies in constructing a heterogeneous graph and applying a depth-first search algorithm to traverse all paths between miRNAs and diseases.

### Matrix factorization-based methods

Over the past decade, matrix factorization and matrix completion have emerged as effective tools for predicting potential MDAs as well as for addressing a wide range of bioinformatics tasks. In this study, we categorize both matrix factorization and matrix completion approaches under the MF-based methods. The core idea of MF-based methods is to project the MDA matrix into a low-dimensional latent space and to infer unobserved associations by completing the missing entries using the learned latent representations.

The development of these methods has evolved along a clear trajectory. Early studies primarily relied on traditional low-rank matrix factorization or nuclear norm minimization techniques, treating the MDA matrix as a sparse matrix to be completed and uncovering latent relationships through simple low-dimensional embeddings [51]. As the field advanced, researchers gradually incorporated prior biological knowledge, such as miRNA similarity and disease similarity, by introducing regularization terms [52–54] or orthogonal constraints [53] to ensure that the factorization process better reflects biological structures. Subsequently, a variety of extended formulations were proposed, including nonnegative matrix factorization (NMF) [34, 55], nonnegative tri-factorization [53], and tensor factorization [52], enabling more flexible modeling of complex relationships.

These methods share several advantages: they can effectively extract hidden low-dimensional structures from highly sparse association data, exhibit strong scalability and flexibility, and can seamlessly integrate diverse biological priors to enhance the inference of potential MDAs. However, their performance typically depends on the low-rank assumption and remains sensitive to data sparsity, making it difficult for such models to capture complex higher order or nonlinear relationships. In this section, we systematically review eight high-impact MF-based methods published between 2018 and 2025 (see Table 3).

**Table 3:** Matrix factorization-based methods for predicting MDAs

Method	Description	Evaluation	Datasets	AUC values	Source Code
**GRNMF** (Xiao et al., 2018) [34]	• Graph regularization; • NMF	five-fold CV	HMDD v2.0	0.869	$^\dagger $ https://github.com/XIAO-HN/GRNMF
**IMCMDA** (Chen et al., 2018) [56]	• Matrix completion; • Integrates MFS, DSS, and GIPK	LOOCV	HMDD v2.0	0.8034	https://github.com/IMCMDAsourcecode/IMCMDA
**MDHGI** (Chen et al., 2018) [57]	• Matrix factorization; • Heterogeneous graph inference; • Sparse learning	five-fold CV, LOOCV	HMDD v2.0	0.8794	https://github.com/wengelearning/MDHGI
**NMFMC** (Zheng et al., 2022) [55]	• NMF; • Matrix completion; • Graph regularization constraints	LOOCV	HMDD v2.0/v3.0	0.9165 (global LOOCV), 0.8512 (local LOOCV)	https://git.l3s.uni-hannover.de/dong/simplifying_mi{RNA}_disease
**WeightTDAIGN** (Ouyang et al., 2022) [52]	• Weighted tensor factorization; • Graph Laplacian regularization; • $ L_{2,1} $ norm constraint	Five-fold CV	HMDD v2.0/v3.2	0.9734 (MDA v2.0), 0.9687 (MDA v3.2)	Unavailable
**SNMDA** (Liu et al., 2024) [58]	• SVD; • Node2vec for nonlinear embeddings; • Gradient Boosting classifier	five-fold CV	HMDD v2.0	0.9608	https://github.com/xiaoyanzi1124/SNMDA.git
**SPLHRNMTF** (Ouyang et al., 2024) [53]	• Robust orthogonal non-negative Matrix tri-factorization; • Self-paced learning; • Dual hypergraph regularization; • $ L_{2,1} $ norm	five-fold CV	HMDD v3.2_data1, HMDD v3.2_data2	0.9304 (v3.2_data1), 0.9358 (v3.2_data2)	https://github.com/Ouyang-Dong/SPLHRNMTF
$ L_{2,1} $ **S-NPFM** (Xie et al., 2025) [54]	• $ L_{2,1} $ similarity constraint graph matrix factorization; • Network projection fusion	five-fold CV, LOOCV	HMDD v2.0	0.9705 (five-fold CV), 0.9723 (LOOCV)	Unavailable

$^\dagger $ “Source Code” is derived from the corresponding paper. “Unavailable” indicates that the “Source Code” is not provided in the corresponding paper. “AUC” is the area under the receiver operating characteristic curve. LOOCV AUC values in the table default to global LOOCV.

**I. Graph regularized nonnegative matrix factorization (GRNMF)** Xiao *et al.* [34] proposed GRNMF for MDA prediction under cold-start scenarios. The core method incorporates DSS and MFS. A weighted *K*-nearest neighbor profile (WKNNP) with decay factor $ \alpha =0.5 $ and neighborhood size $ K=5 $ reconstructs sparse entries in the adjacency matrix $ Y $. The graph-regularized NMF objective function is formulated as


(3) \begin{align*}& \begin{split} \min_{W,H} \ & \|Y - WH^{T}\|_{F}^{2} + \lambda_{l}\big(\|W\|_{F}^{2} + \|H\|_{F}^{2}\big) \\ & + \lambda_{m} \mathrm{Tr}(W^{T} L_{m} W) + \lambda_{d} \mathrm{Tr}(H^{T} L_{d} H) \end{split},\end{align*}

where $ L_{m} = D_{m} - S_{m}^{*} $ and $ L_{d} = D_{d} - S_{d}^{*} $ are graph Laplacians constructed from $ p $ nearest neighbors and ClusterONE clusters. $\lambda _{l}$, $\lambda _{m}$, and $\lambda _{d}$ are the regularization coefficients. Matrices $ W \in \mathbb{R}^{n \times k} $ and $ H \in \mathbb{R}^{m \times k} $ are optimized via alternating updates until convergence, generating the predicted association matrix $Y^{*} = WH^{T}$.

**Figure 5:**
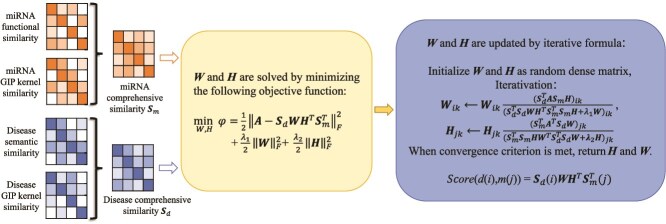
Flowchart of IMCMDA model to infer the potential MDAs.


**II. Inductive matrix completion for MDA prediction (IMCMDA)** Chen *et al.* [56] proposed IMCMDA to address cold-start challenges in MDA prediction as shown in Fig. 5. The core idea of the IMCMDA model is to transform the sparse association matrix completion problem into a constrained low-rank matrix factorization optimization problem by fusing disease and miRNA similarity features, and finally obtain the association prediction scores through iterative solution. The model incorporates DSS, MFS, and GIPK similarity. The inductive matrix completion (IMC) method completes the missing elements (i.e. predicts unknown MDAs) in the association matrix $ A $ and finally obtains a complete prediction matrix $ Z $, where the elements represent the scores of the association probability. Assume that the completed matrix $ Z $ can be decomposed into low-rank matrices $ W $ and $ H $. $ W $ and $ H $ are solved by minimizing the following objective function:

(4) \begin{align*}& \underset{W, H}{\min} \quad \Phi = \frac{1}{2} \left\| A - S_{d} W H^{T} S_{m}^{T} \right\|_{F}^{2} + \frac{\lambda_{1}}{2} \| W \|_{F}^{2} + \frac{\lambda_{2}}{2} \| H \|_{F}^{2},\end{align*}

where $S_{d}$ is the disease similarity matrix, and $S_{m}$ is the miRNA similarity matrix. $W$ and $H$ are the low-rank matrices obtained from matrix factorization. The Frobenius norm $\| \cdot \|_{F}$ is used to measure the magnitude of matrices. $ \lambda _{1} $, $ \lambda _{2} $ are the regularization coefficients. $ W $ and $ H $ are alternately updated through iterative formulas (see Fig. 5 for specific steps) until the convergence condition is met, and the optimal $ W $ and $ H $ are obtained. To make the decomposition results more consistent with biological characteristics, the model introduces disease and miRNA similarity matrices as constraints, and modifies Z to $Z = S_{d} W H^{T} S_{m}^{T}$.

The association possibility score between disease $d(i)$ and miRNA $m(j)$ is calculated by the following formula:


(5) \begin{align*}& \mathrm{Score}(d(i), m(j)) = S_{d}(i) \, W \, H^{T} \, S_{m}^{T}(j),\end{align*}

where $S_{d}(i)$ is the feature vector of disease $d(i)$, and $S_{m}^{T}(j)$ is the feature vector of miRNA $m(j)$. A higher score indicates a greater possibility of association between them.

**III. Matrix decomposition and heterogeneous graph inference for MDA prediction (MDHGI)** Chen *et al.* [57] proposed MDHGI to address noise handling and cold-start challenges in MDA prediction. The method integrates matrix decomposition with heterogeneous graph inference, leveraging sparse learning to decompose the known association matrix into a low-rank matrix and a sparse noise matrix. The reconstructed matrix $ A^{*} $ is combined with MFS, DSS, and GIPK similarity to build a heterogeneous graph. Association probabilities are computed iteratively.

**IV. Matrix completion algorithm based on nonnegative matrix factorization (NMFMC)** Zheng *et al.* [55] proposed NMFMC to solve the sparsity issue by integrating NMF with matrix completion for MDA prediction. The core innovation lies in decomposing the known association matrix into a known part and an unknown part, and approximating the complete matrix through NMF. Graph regularization is introduced to preserve the local geometric structure of data, ensuring that functionally similar miRNAs are more likely associated with phenotypically similar diseases.

**V. Weighted tensor decomposition with auxiliary information, graph Laplacian regularization, and $ L_{2,1} $ norm (WeightTDAIGN)** Ouyang *et al.* [52] proposed the WeightTDAIGN to address the challenge of predicting multiple types of MDAs. The model integrates weighted tensor decomposition with graph Laplacian regularization and the $ L_{2,1} $ norm to improve prediction performance. The core innovation lies in the introduction of a weighting mechanism that assigns higher weights to positive samples to enhance their recovery and overall prediction accuracy. WeightTDAIGN incorporates various auxiliary information related to miRNAs and diseases, such as MSS, DFS, and DSS. It also employs graph Laplacian regularization to preserve the local structure of biological similarity networks and uses the $ L_{2,1} $ norm to constrain projection matrices, thereby reducing the impact of noise on the model.

**VI. Singular value decomposition and Node2vec for MDA prediction (SNMDA)** Liu *et al.* [58] proposed SNMDA to combine singular value decomposition (SVD) and node2vec for predicting MDAs. The model extracts linear features from the MDA matrix using SVD, capturing essential linear associations, while node2vec learns nonlinear embeddings from integrated similarity networks of miRNAs and diseases. These linear and nonlinear features are fused to form comprehensive feature vectors, which are then fed into a gradient boosting (GB) classifier for binary classification.

**Figure 6:**
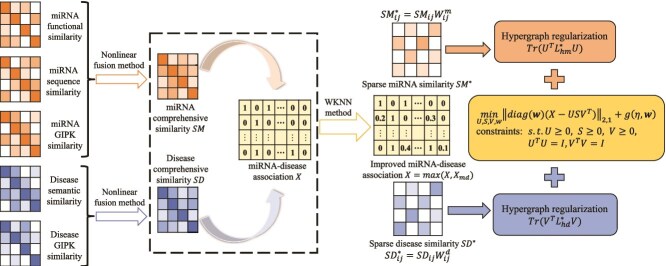
Flowchart of the SPLHRNMTF model, which combines self-paced learning (SPL), hypergraph regularization, and NMTF to infer potential MDA. In the formula, $X_{md}$ denotes the new interaction profiles between miRNAs and diseases, which are computed using the WKNN method. The k-nearest neighbor weighted matrices $W_{m}^{ij}$ and $W_{d}^{ij}$ can be calculated based on the miRNA comprehensive similarity matrices $SM$ and disease comprehensive similarity matrices $SD$, respectively. $\mathrm{Tr}(\cdot )$ represents the trace of a matrix (the sum of elements on the main diagonal, used to convert matrix operations into scalar loss). $U$ denotes the miRNA low-dimensional embedding matrix, $S$ denotes the factor matrix, and $V$ denotes the disease low-dimensional embedding matrix. $\mathbf{w}$ represents the sample weight vector for SPL, where $\mathrm{diag}(\mathbf{w})$ is used to convert $\mathbf{w}$ into a diagonal matrix. $L^{*}_{hm}$ refers to the miRNA hypergraph Laplacian matrix, and $L^{*}_{hd}$ refers to the disease hypergraph Laplacian matrix. $g(\eta , \mathbf{w})$ is the regularization term of SPL, and $\|\cdot \|_{2,1}$ denotes the $ L_{2,1} $ norm of a matrix.

**VII. Robust orthogonal nonnegative matrix tri-factorization with self-paced learning and dual hypergraph regularization (SPLHRNMTF)** Ouyang *et al.* [53] proposed SPLHRNMTF (see Fig. 6), whose core methodology involves a nonlinear fusion strategy to combine MFS, DSS, and GIPK similarity into comprehensive miRNA and disease similarity networks. The model refines the MDA matrix using weighted *k*-nearest neighbor profiles to address false-negative samples. To enhance robustness, SPLHRNMTF replaces the Frobenius norm with the $ L_{2,1} $ norm for residual error calculation, reducing the impact of noise and outliers. Self-paced learning (SPL) is incorporated into the nonnegative matrix tri-factorization (NMTF) method to gradually include samples from easy to complex, preventing the model from falling into poor local optima. Hypergraph regularization is employed to capture high-order complex associations within miRNA and disease similarity networks, enhancing low-dimensional embeddings.

**VIII. Integrating $ L_{2,1} $-norm constraint and network projection fusion for MDA prediction ($ L_{2,1} $-NPFM)** Xie *et al.* [54] proposed $ L_{2,1} $S-NPFM, a method combining $ L_{2,1} $-norm constrained matrix factorization ($ L_{2,1} $SGMF) and network projection fusion (NPFM) to predict MDAs. The $ L_{2,1} $SGMF module introduces a similarity constraint based on the $ L_{2,1} $-norm to suppress noise in low-dimensional matrices, while the NPFM module integrates miRNA/disease similarity networks with the initial score matrix to compensate for lost network topology information during decomposition. The final score matrix is obtained by fusing the network projection matrices back into the initial score matrix.

### Machine learning-based methods

Over the past decade, ML-based methods have achieved substantial progress, with an increasing number of machine learning models proposed to extract more informative features and thereby improve predictive performance. Generally, ML-based approaches formulate MDA prediction as a supervised or semi-supervised learning task. Their core idea is to construct high-dimensional feature vectors from miRNA similarity, disease similarity, and heterogeneous relational networks through feature engineering or automated feature representation, and then train classifiers or regression models to infer previously unobserved associations.

The development of these methods has followed a clear evolution. Early studies relied on traditional machine learning models such as RLS [23, 59, 60] and multi-kernel learning [59]. Subsequent work shifted toward dimensionality-reduction techniques, including principal component analysis (PCA) [61] and autoencoders [62], combined with ensemble learning models such as random forests [63–65] and gradient boosting trees [66] to enhance representation capacity and capture more informative patterns.

These methods share several advantages: they are computationally efficient, easy to implement, and offer fast training speed with relatively low computational cost. However, ML-based approaches usually depend on predefined feature extraction and cannot be trained in an end-to-end manner, which limits their ability to capture complex nonlinear and high-order interactions. In this section, we systematically reviewed 11 high-impact ML-based studies published between 2014 and 2025 (see Table 4), categorizing them into four main categories.

**Table 4:** Machine learning-based methods for predicting MDAs

Method	Description	Evaluation	Datasets	AUC values	Source Code
**RLSMDA** (Chen et al., 2014) [23]	• RLS; • Semi-supervised learning; • Global prediction for all diseases simultaneously	LOOCV	HMDD v2.0	0.9511 (GL), 0.8450 (LL)	Unavailable
**MKRMDA** (Chen et al., 2017) [59]	• Multi-kernel learning; • Kronecker regularized least squares; • Two-step optimization for kernel combination	five-fold CV, LOOCV	HMDD v2.0	0.8894 (five-fold CV), 0.9040 (GL), 0.8446 (LL)	http://www.cuilab.cn/files/images/cuilab/misim.zip
**KRLSM** (Luo et al., 2017) [60]	• Comprehensive similarity measures using heterogeneous omics data; • Kronecker product to combine miRNA and disease spaces; • Regularized least squares classifier	five-fold CV	HMDD v2.0	0.853	$^\dagger $ https://github.com/XIAO-HN/KRLSM
**RFMDA** (Chen et al., 2018) [63]	• Random Forest-based prediction; • Integrates MFS, DSS, and GIPK similarity	five-fold CV, LOOCV	HMDD v2.0	0.8818 (five-fold CV), 0.8891 (GL), 0.8323 (LL)	Unavailable
**EGBMMDA** (Chen et al., 2018) [66]	• Extreme Gradient Boosting Machine; • Feature engineering with MFS, DSS, and matrix factorization results	five-fold CV, LOOCV	HMDD v2.0	0.9048 (five-fold CV), 0.9123 (GL), 0.8221 (LL)	Unavailable
**IRFMDA** (Yao et al., 2019) [64]	• Integrated similarity of diseases and miRNAs; • Feature selection based on RF variable importance score; • Random Forest regression model	five-fold CV, LOOCV	HMDD v2.0	0.9363 (five-fold CV), 0.9398 (GL), 0.8728 (LL)	Unavailable
**Ji’s method** (Ji et al., 2020) [65]	• Heterogeneous information network construction; • GraRep for node embedding; • Random Forest classifier	five-fold CV	HMDD v3.0	0.9125	$^\dagger $ https://github.com/jiboya123/working-code.git
**MDA-CF** (Dai et al., 2021) [62]	• Cascade forest model; • Multi-source information fusion; • Autoencoder for dimension reduction	five-fold CV	HMDD v2.0/v3.2	0.9258 (v2.0), 0.9464 (v3.2)	https://github.com/a1622108/MDA-CF
**ELMDA** (Gu et al., 2023) [67]	• Ensemble learning framework; • Multi-classifiers voting; • miRNA and disease similarity integration	five-fold CV	HMDD v2.0	0.9229	https://github.com/Changlong2020/ELMDA
**PCACFMDA** (Zhang et al., 2024) [61]	• Fusion of miRNA and disease similarities; • PCA for dimensionality reduction; • Optimized cascade forest for prediction	five-fold CV, 10-fold CV	HMDD v2.0	0.9856 (five-fold CV), 0.9858(10-fold CV)	https://github.com/zhtdbobo/PCACFMDA
**DWMDA** (Ha et al., 2025) [68]	• DeepWalk-based graph embedding; • Low-dimensional vector representation; • Deep neural network for classification	LOOCV	HMDD v3.2	0.9260 (GL), 0.9101 (LL)	Unavailable

“Unavailable” indicates that the “Source Code” is not provided in the corresponding paper. “AUC” is the area under the receiver operating characteristic curve. LOOCV AUC values in the table default to global LOOCV. “GL” represents global LOOCV. “LL” represents local LOOCV.

#### Random forest

**I. Random forest for MDA prediction (RFMDA)** Chen *et al.* [63] proposed RFMDA, a RF-based model for MDA prediction. The method integrates MFS, DSS, and GIPK similarity to construct feature vectors for miRNA–disease pairs. Positive and negative samples were selected from known associations in HMDD v2.0 and randomly paired unknown associations, respectively. Each miRNA–disease pair was represented by a feature vector combining miRNA and disease similarity profiles, followed by dimensionality reduction to 100 discriminative features. The model is trained using the RF algorithm to generate prediction scores, where higher scores indicate a greater likelihood of association.

**II. Improved random forest-based model for MDA prediction (IRFMDA)** Yao *et al.* [64] proposed IRFMDA, an improved computational method based on RF for predicting MDAs. The method integrates DSS, GIPK similarity, and MFS to represent miRNA–disease pairs. Feature selection based on RF variable importance scores is then performed to optimize the model’s ability to infer MDAs.

**III. GraRep embedding model for MDA prediction (Ji’s method)** Ji *et al.* [65] proposed a heterogeneous information network method based on the GraRep embedding model to predict MDAs. The method constructs a heterogeneous information network by integrating known associations among lncRNA, drugs, proteins, diseases, and miRNAs. The GraRep method is then employed to learn the behavior information of nodes in the network. The embedding representations of miRNA and disease are generated by integrating their attribute information (e.g. MSS and DSS) with the learned behavior information. Finally, a RF classifier is used to predict potential MDAs.

#### XGBoost

Chen *et al.* [66] proposed EGBMMDA, which integrates MFS, DSS, and known MDAs to construct a comprehensive feature vector, which includes statistical measures, graph-theoretical metrics, and matrix factorization results. Gu *et al.* [67] proposed ELMDA, an ensemble learning method for MDA prediction, particularly addressing cold-start scenarios. The method integrates MFS and DSS to construct comprehensive similarity networks. ELMDA employs a multi-classifier voting mechanism (SVM, GBDT, RF, XGBoost) to predict potential MDAs.

#### Regularized least square

**I. Multiple kernel learning-based kronecker regularized least squares for MDA prediction (MKRMDA)** Chen *et al.* [59] proposed MKRMDA, a computational method for MDA prediction based on multiple kernel learning and kronecker RLS. The method integrates various data sources, including MFS, DSS, and GIPK similarity, and employs a two-step optimization process to automatically optimize the combination of multiple kernels. This approach effectively leverages multi-source data to improve prediction accuracy.

**II. Kronecker regularized least squares for MDA prediction (KRLSM)** Luo *et al.* [60] proposed KRLSM, which integrates multi-omics data and kronecker regularized least squares (KRLS). The method innovatively combines DSS and MFS to construct a comprehensive miRNA–disease space using the kronecker product. A semi-supervised classifier is then employed to identify disease-related miRNAs.

#### Others

**Figure 7:**
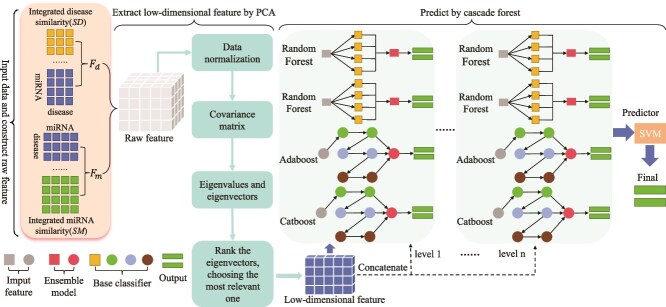
The framework of PCACFMDA model. $F_{m}$ and $F_{d}$ represent the integrated feature matrix for miRNA and disease, respectively.

Xing *et al.* [23] proposed RLSMDA, a semi-supervised learning method for predicting potential MDAs. This method constructs RLS by integrating known MDAs, DSS, and MFS, thereby overcoming the traditional method’s reliance on negative samples and being able to predict potential miRNA associations for all diseases at the same time. RLSMDA can predict miRNAs for diseases without any known associated miRNAs. Dai *et al.* [62] proposed MDA-CF to predict MDAs by integrating multisource information (including MSS, MFS and DSS). The method employs an autoencoder to reduce feature dimensions to 128, extracting an optimal feature space for miRNAs and diseases. The cascade forest model combines RF and XGBoost in a layered structure. The model complexity is automatically determined during training, avoiding overfitting. Ha *et al.* [68] proposed DWMDA, a method for predicting MDAs by integrating deepwalk graph embedding and DNNs. The method utilizes deepwalk to extract low-dimensional vectors from miRNA and disease networks and employs a DNN to identify potential MDAs. Zhang *et al.* [61] proposed the PCACFMDA (see Fig. 7), which integrates similarities of miRNAs and diseases, and known MDAs, to construct a multidimensional feature matrix. PCA is then applied to reduce data complexity and extract low-dimensional features. Subsequently, an optimized cascade forest is used to deeply mine the features and output prediction scores.

### Non-graph deep learning-based methods

The core idea of non-graph deep learning methods (DL-based methods) is to automatically learn high-dimensional nonlinear representations of miRNAs and diseases in an end-to-end manner through DNNs, thereby enabling the prediction of their potential associations. The development of these methods has followed a clear trajectory: early studies primarily employed fully connected neural networks to perform nonlinear mapping of miRNA and disease similarity features, while more recent work has introduced deeper architectures, such as autoencoder [69–71], convolutional neural network (CNN) [72, 73], multilayer perceptron (MLP) [74], and attention mechanisms [41], to integrate multisource biological information and automatically extract informative embedding representations.

These methods share several advantages: they can autonomously learn high-dimensional nonlinear representations from heterogeneous biological data, effectively capture complex latent relationships between miRNAs and diseases, and substantially improve predictive performance without requiring extensive feature engineering. However, DL-based methods are inherently incapable of modeling graph-structured data and thus overlook the interactions between nodes. In this section, we systematically reviewed 14 high-impact DL-based methods published in recent years (see Table 5), which are divided into three categories.

**Table 5:** Non-graph deep learning-based methods for predicting MDAs

Method	Description	Evaluation	Datasets	AUC values	Source Code
**DeepMDA** (Fu et al., 2017) [69]	• Deep ensemble model using stacked autoencoders; • High-order feature extraction from miRNA and disease similarity data; • Three-layer neural network for prediction	five-fold CV,LOOCV	HMDD v2.0	0.9486 (five-fold CV), 0.8729 (GL)	https://github.com/sperfu/DeepMDA
**CNNDMP** (Xuan et al., 2018) [73]	• Dual CNN; • RWR for network topology; • Integration of original features and network topology	five-fold CV	HMDD v2.0	0.956	Unavailable
**CNNMDA** (Xuan et al., 2019) [75]	• Network representation learning; • Dual CNNs; • NMF	five-fold CV	HMDD v2.0	0.968	Unavailable
**EPMDA** (Dong et al., 2019) [74]	• Edge perturbation; • Feature vector with structural Hamiltonian information; • MLP	five-fold CV, LOOCV	HMDD v2.0	0.9818 (five-fold CV), 0.9371 (LOOCV)	https://github.com/DYDGitHub/EPMDA
**MDA-CNN** (Peng et al., 2019) [72]	• Three-layer network feature extraction; • Auto-encoder feature selection; • CNN prediction	10-fold CV	HMDD v2.0	0.8897	https://github.com/Issingjessica/MDA-CNN
**AEMDA** (Ji et al., 2021) [70]	• Learning-based method for dense representation; • Deep autoencoder without negative samples; • Reconstruction error for prediction	five-fold CV, LOOCV	HMDD v2.0	0.9383 (five-fold CV), 0.9410 (GL)	https://github.com/CunmeiJi/AEMDA
**SMALF** (Liu et al., 2021) [25]	• Stacked autoencoder for latent feature extraction; • Integration of MFS, DSS, and latent features; • XGBoost for prediction	five-fold CV	HMDD v2.0	0.9503	https://github.com/dayunliu/SMALF
**DANE-MDA** (Ji et al., 2021) [76]	• Deep attributed network embedding; • Fusion of structure and attribute features; • Deep stacked auto-encoder for high-order feature extraction	five-fold CV	HMDD v2.0/v3.0	0.9264 (HMDD v2.0), 0.9113 (HMDD v3.0)	$^\dagger $ https://github.com/jiboya123/DANE-MDA
**MvKFN-MDA** (Li et al., 2021) [77]	• Multi-view kernel fusion network; • Neural matrix completion for prediction; • Nonlinear multiple kernels fusion	five-fold CV, LOOCV	HMDD v2.0	0.9383 (five-fold CV), 0.9591 (GL)	$^\dagger $ https://github.com/JinLi-YNU/MvKFN-MDA
**MLRDFM** (Ding et al., 2022) [78]	• Multi-view Laplacian regularized DeepFM; • Integrates Laplacian regularization and Laplacian eigenmap initialization	five-fold CV	HMDD v3.2	0.9545	https://github.com/XYDBCS/MLRDFM
**SAEMDA** (Wang et al., 2022) [79]	• Stacked autoencoder pretraining; • Fine-tuning with softmax classifier; • Utilizes feature information of unlabeled pairs	five-fold CV, LOOCV	HMDD v2.0	0.9102 (five-fold CV), 0.9210 (GL), 0.8343 (LL)	https://github.com/xpnbs/SAEMDA
**DNRLCNN** (Zhong et al., 2022) [80]	• Latent feature matrix extraction using only positive samples; • Feature vector construction for miRNA–disease pairs; • Modified CNN for prediction	five-fold CV	HMDD v3.2	0.9030 (miRNA–disease), 0.9442 (miRNA-phenotype)	Unavailable
**DFELMDA** (Liu et al., 2022) [71]	• New feature representation strategy; • Deep autoencoders for low-dimensional feature extraction; • Deep forest ensemble learning for prediction	five-fold CV, 10-fold CV	HMDD v2.0	0.9552 (five-fold CV), 0.9560(10-fold CV)	https://github.com/Zj-Teng/DFELMDA
**PATMDA** (Xie et al., 2023) [41]	• Positive PPMI; • Attention network; • RWR; • CNN	five-fold CV	HMDD v2.0/v3.2	0.933 (HMDD v2.0), 0.946 (HMDD v3.2)	https://github.com/xxpaaa/PATMDA

$^\dagger $ “Source Code” is derived from the corresponding paper. “Unavailable” indicates that the “Source Code” is not provided in the corresponding paper. “AUC” is the area under the receiver operating characteristic curve. “GL” represents global LOOCV. “LL” represents local LOOCV. “miRNA-phenotype” represents the miRNA-phenotype association prediction task.

#### Autoencoder

**I. A Deep ensemble model for MDA prediction (DeepMDA)** Fu *et al.* [69] proposed DeepMDA, which integrates MFS, known MDAs, and DSS to construct miRNA–miRNA and disease–disease similarity networks. DeepMDA employs SAEs to extract high-order features from similarity matrices and utilizes a three-layer fully connected neural network for prediction. The core innovation lies in combining GIPK similarity with DSS, leveraging SAEs to capture nonlinear patterns and reducing reliance on known MDAs.

**II. Deep autoencoder for MDA prediction (AEMDA)** Ji *et al.* [70] proposed AEMDA, which integrates DSS, MFS, and GIPK similarity to construct high-order representations of diseases and miRNAs. AEMDA consists of two key components: regression models to learn dense vector representations for diseases and miRNAs. A seven-layer deep autoencoder that captures latent associations from known MDAs. The autoencoder minimizes the reconstruction error, where smaller errors indicate stronger associations.

**III. Stacked autoencoder and XGBoost for MDA prediction (SMALF)** Liu *et al.* [25] proposed SMALF, which extracts latent features from the original MDA matrix using stacked autoencoders, which minimize reconstruction error and jacobian regularization terms. These latent features are combined with MFS and DSS to construct feature vectors for miRNA–disease pairs. The fused features are classified by XGBoost, whose objective function optimizes model complexity through gradient-boosted trees.

**IV. Deep attributed network embedding for MDA prediction (DANE-MDA)** Ji *et al.* [76] proposed DANE-MDA, which integrates network structure and attribute features by leveraging a deep stacked auto-encoder to extract high-order representations from diverse proximity matrices. The method first computes disease and miRNA structural and attribute features, then captures interactions between these features through personalized RWs to construct an enhanced matrix representation that preserves both local and global network structures. A deep stacked auto-encoder is employed to learn complex nonlinear patterns from the enhanced matrix, generating low-dimensional embeddings for miRNAs and diseases.

**V. Stacked autoencoder for MDA prediction (SAEMDA)** Wang *et al.* [79] proposed SAEMDA, which integrates MFS, DSS, and GIPK similarity to construct feature vectors for all miRNA–disease pairs. SAEMDA employs a two-stage training strategy: unsupervised pretraining of the stacked autoencoder using all miRNA–disease pairs to learn low-dimensional representations; and supervised fine-tuning with an added softmax output layer using positive samples (known MDAs) and an equal number of randomly selected negative samples (unlabeled pairs). The model consists of three hidden layers with tanh activation functions, optimized via Adam optimizer and cross-entropy loss. This approach effectively leverages unlabeled data to enhance prediction accuracy in scenarios with limited labeled samples.

**VI. Deep forest ensemble learning based on autoencoder (DFELMDA)** Liu *et al.* [71] proposed DFELMDA, to address the challenges of high-dimensional data and sample sparsity in MDA prediction. The method integrates MFS, DSS, and GIPK similarity, constructing dual-perspective feature representations from both miRNA and disease views. Two deep autoencoders are employed to extract low-dimensional feature representations, followed by RF classifiers to predict associations from each perspective. The final prediction score is obtained by weighted fusion of the two RF outputs. Key innovations include the dual-perspective feature representation strategy and the use of autoencoders for dimensionality reduction, which effectively handle the high-dimensional and sparse nature of the data.

#### Convolutional neural networks

**I. Dual Convolutional Neural Networks for MDA prediction (CNNDMP)** Xuan *et al.* [73] proposed CNNDMP model. By integrating the original features of miRNAs and diseases as well as their network topological structures, CNNDMP leverages a dual CNN for efficient prediction. This method innovatively combines the original similarities and associations of miRNAs and diseases. It obtains novel similarities containing topological structures through RWs and utilizes the deep feature extraction capability of CNN to tackle the challenges of sparse data and deep feature mining faced by traditional methods.

**II. Network representation learning and Convolutional Neural networks for MDA prediction (CNNMDA)** Xuan *et al.* [75] proposed CNNMDA, which integrates the similarity information between miRNA and disease, known MDA and its representation in low-dimensional feature space, and uses network representation learning and CNN for prediction. This method innovatively combines these elements and uses a deep learning framework to learn the original and global representations of miRNA–disease pairs.

**III. A learning-based method for MDA prediction (MDA-CNN)** Peng *et al.* [72] proposed MDA-CNN, which constructs a three-layer network integrating disease similarity, miRNA similarity, and protein–protein interaction networks to extract interaction features for miRNA–disease pairs. An auto-encoder is employed to reduce the dimensionality of high-dimensional feature vectors, identifying essential feature combinations, while CNN is utilized to learn the optimal feature representations and predict MDAs. The key innovation lies in the introduction of a gene-layer network, which captures deep interaction patterns between miRNAs and diseases, addressing the limitations of traditional methods that rely solely on direct associations.

**IV. A CNN method for MDA prediction (DNRLCNN)** Zhong *et al.* [80] proposed DNRLCNN, to address the uncertainty of negative samples in MDA prediction. The method integrates latent feature extraction using only positive samples with CNN. The core algorithm combines logistic matrix factorization and CNN, constructing low-dimensional latent feature matrices $ U $ and $ V $ through the optimization objective:


(6) \begin{align*} \begin{split} \min_{(U, V)} L = &\sum_{i=1}^{N_{m}} \sum_{j=1}^{N_{d}} r_{ij=1} \left[ r_{ij} \ln \left( 1 + \exp \left( u_{i} v_{j}^{T} \right) \right) - r_{ij} u_{i} v_{j}^{T} \right] \\ &+ \frac{1}{2} \operatorname{tr} \left( U^{T} \left( \lambda_{m} I + \alpha L^{m} \right) U \right) \\ &+ \frac{1}{2} \operatorname{tr} \left( V^{T} \left( \lambda_{d} I + \beta L^{d} \right) V \right), \end{split}\end{align*}

where $\lambda _{m}$, $\lambda _{d}$, $\alpha $, $\beta $, and $r$ are hyperparameters. $\alpha $ and $\beta $ are used to set the weight of the dynamic nearest neighbor matrix $A$ and $B$, respectively. $r$ is the number of dimensions of the latent feature matrix for complex interactions between miRNAs and diseases in low-dimensional space. $U \in \mathbb{R}^{r \times N_{m}}$ and $V \in \mathbb{R}^{r \times N_{d}}$ represent the set of all potential vectors for miRNAs and diseases. $u_{i} \in \mathbb{R}^{r \times 1}$ and $v_{j} \in \mathbb{R}^{r \times 1}$ denote the low-dimensional potential vectors of miRNA $m_{i}$ and disease $d_{j}$, respectively. $L^{m}$ and $L^{d}$ are the Laplacian matrices for miRNAs and diseases, respectively. $I$ represents the identity matrix. $\operatorname{tr}(\cdot )$ denotes the trace of a matrix.

**V. Positive point-wise mutual information and attention network to predict MDAs (PATMDA)** Xie *et al.* [41] proposed PATMDA, which integrates positive point-wise mutual information (PPMI) and an attention network. The method constructs a heterogeneous MDA network and multiple similarity networks (including DSS, MFS, and GIPK similarity). Multi-order proximity features are extracted from different similarity views using RWR and PPMI, and then use CNN to fuse these features and generate high-order proximity representations. An attention network with neural aggregation is designed to integrate node representations and their heterogeneous neighbors based on the MDA network. The inner product decoder was used to calculate the association scores.

#### Others

**Figure 8:**
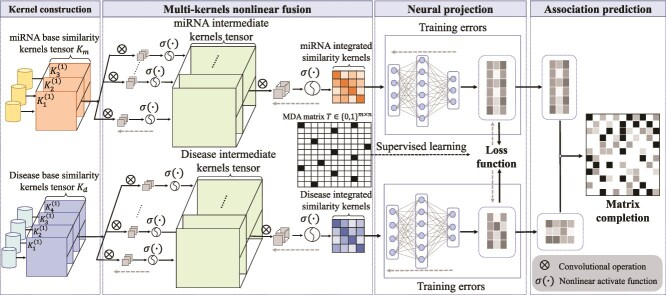
The framework of MvKFN-MDA model. $K_{1}^{(1)}, K_{2}^{(1)}, K_{3}^{(1)}$, and $K_{4}^{(1)}$ denote the similarity kernels for view 1, view 2, view 3, and view 4, respectively.

Dong *et al.* [74] proposed EPMDA, a method for MDA prediction based on edge perturbation and MLP. A unique feature extraction method is designed by measuring the impact of edge perturbations on the structural Hamiltonian, which quantifies the global structural changes caused by adding or removing edges. These features are used to train an MLP model for predicting potential MDAs. Li *et al.* [77] proposed MvKFN-MDA (see Fig. 8), which integrates multi-view similarity kernels (including MSS, MFS, DSS and GIPK similarity) using a nonlinear multi-view kernel fusion network. The integrated kernels are input into a neural matrix completion model to extract feature representations of miRNAs and diseases, ultimately outputting association prediction scores.

**Figure 9:**
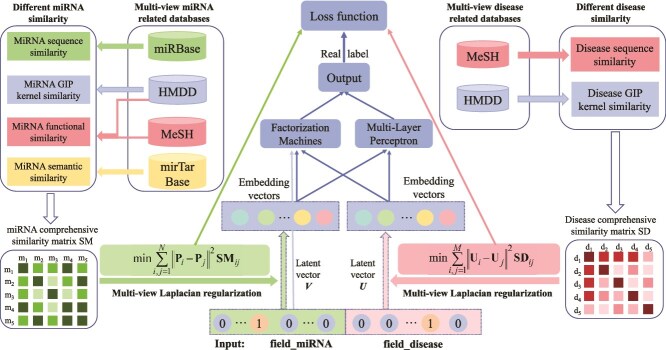
The framework of MLRDFM model. The left component corresponds to Laplacian regularization based on multi-view miRNA similarity, the right component denotes Laplacian regularization based on multi-view disease similarity, and the central component is a DeepFM model. In the original paper, $\mathbf{P}_{i}$ and $\mathbf{P}_{j}$ are assumed to be the column latent vectors for miRNAs $m_{i}$ and miRNA $m_{j}$, respectively. $\mathbf{U}_{i}$ and $\mathbf{U}_{j}$ are the latent vectors of disease $i$ and disease $j$, respectively.

Ding *et al.* [78] proposed MLRDFM (see Fig. 9), which improved the deep factorization machine (DeepFM) model with multi-view Laplacian regularization. The method introduces two Laplacian regularizations to constrain the weights in the embedding layers of miRNAs and diseases, respectively, and employs Laplacian eigenmap initialization to avoid local minima in training. The model integrates multi-view similarity information to enhance the robustness of the prediction.

### Graph neural network-based methods

The core idea of GNN-based methods for MDA prediction is to perform message passing and node representation learning on heterogeneous networks that integrate multiple biological similarities and known associations. By propagating and aggregating information across the network, these models can automatically capture higher-order topological relationships between miRNAs and diseases, thereby enabling accurate inference of unobserved associations.

The development of this line of research has shown a clear trajectory: early studies primarily relied on GCNs [26, 81–83] or graph autoencoders (GAEs) [84–86] to learn node embeddings from miRNA–disease heterogeneous networks. More recent work has advanced toward hypergraph convolutional networks (HGCNs) [33, 87–89], which are designed to capture richer high-order relationships and encode more complex network topology, ultimately improving prediction accuracy.

These methods share the advantage of fully exploiting the topological structure and high-order dependencies within miRNA–disease heterogeneous networks, allowing models to automatically learn expressive node representations through message propagation and neighbor aggregation. However, GNN-based methods are sensitive to the quality and completeness of the heterogeneous network, and their black-box nature imposes limitations on interpretability. In this section, we systematically reviewed 22 high-impact GNN-based studies published in recent years (see Table 6), which are categorized into five categories.

**Table 6:** GNN-based methods for predicting MDAs

Method	Description	Evaluation	Datasets	AUC values	Source Code
**NIMCGCN** (Li et al., 2020) [81]	• GCNs for feature learning; • Neural IMC	five-fold CV, LOOCV	HMDD v2.0	0.9291 (five-fold CV), 0.9387 (LOOCV)	https://github.com/ljatynu/NIMCGCN/
**MMGCN** (Tang et al., 2021) [26]	• Multi-view GCN encoder for miRNA and disease features; • Multichannel attention mechanism; • CNN combiner for final embedding	five-fold CV	HMDD v3.2	0.9266	https://github.com/Txinru/MMGCN
**GAEMDA** (Li et al., 2021) [84]	• GNN-based encoder; • Bilinear decoder for link prediction; • End-to-end training	five-fold CV	HMDD v2.0	0.9356	https://github.com/chimianbuhetang/GAEMDA
**VGAMF** (Ding et al., 2022) [85]	• Variational GAE for nonlinear representation; • Matrix factorization for linear representation; • Fully connected neural network for final prediction	10-fold CV	HMDD v2.0/v3.2	0.9280 (v2.0), 0.9470 (v3.2)	https://github.com/XYDBCS/VGAMF
**AGAEMD** (Zhang et al., 2022) [86]	• Node-level attention encoder-decoder network; • Low-dimensional dense embeddings for node representation; • Heterogeneous matrix integration	five-fold CV	HMDD v2.0/v3.2	0.9154 (v2.0), 0.9261 (v3.2)	https://github.com/Zhhuizhe/AGAEMD
**MDPBMP** (Yu et al., 2022) [90]	• miRNA–disease-gene heterogeneous network; • Feature extraction using symmetrical meta-paths; • Aggregation of node information via multi-head attention	five-fold CV	HMDD v2.0/v3.2	0.9214, 0.9294 (v2.0), 0.9378 (v3.2)	https://github.com/LiangYu-Xidian/MDPBMP
**GRPAMDA** (Zhong et al., 2022) [91]	• Graph random propagation network; • Attention mechanism for feature aggregation; • Fully connected layer for scoring	five-fold CV	HMDD v2.0	0.9346	https://github.com/ZTangBo/GRPAMDA
**HGANMDA** (Li et al., 2022) [92]	• Hierarchical GAT; • Node-layer attention and semantic-layer attention; • Bilinear decoder for MDA prediction	five-fold CV	HMDD v2.0	0.9374	https://github.com/ZTangBo/HGANMDA
**MKGAT** (Wang et al., 2022) [93]	• GATs for feature extraction; • Dual Laplacian regularized least squares for prediction; • Kernel fusion with attention mechanism	five-fold CV	HMDD v2.0	0.9627	https://github.com/shine-lucky/MKGAT-main
**MGCNRF** (Yang et al., 2023) [94]	• Multiple heterogeneous networks; • Layer attention GCNs; • Random forest for prediction	five-fold CV	HMDD v3.2	0.9455	Unavailable
**AMHMDA** (Ning et al., 2023) [87]	• Attention-aware multi-view similarity networks; • Hypergraph learning with hypernodes; • Layer-level attention mechanism	five-fold CV	HMDD v3.2	0.9422	https://github.com/ningq669/AMHMDA
**HGTMDA** (Lu et al., 2024) [42]	• Hypergraph learning; • GCN-Transformer and RWR; • Association masking	five-fold CV	HMDD v3.2	0.9507	Unavailable
**MGCNSS** (Tian et al., 2024) [82]	• Multi-layer graph convolution; • Distance-based negative sample selection; • Meta-path relation learning	five-fold CV	HMDD v2.0	0.9874 (1:1), 0.9861 (1:5), 0.9871 (1:10)	https://github.com/15136943622/MGCNSS/tree/master
**HHOMR** (Li et al., 2024) [95]	• Hybrid high-order moments; • Element-level attention mechanisms; • GNN	five-fold CV	HMDD v2.0	0.9328	https://github.com/W-LP/HHOMR
**MHCLMDA** (Peng et al., 2024) [88]	• Hypergraph contrastive learning; • Hypergraph convolution and multi-view learning; • VAE	five-fold CV	HMDD v3.2	0.9454	https://github.com/weiba/MHCLMDA
**SGLMDA** (Ji et al., 2024) [96]	• Subgraph learning based on heterogeneous networks; • GNN-based feature extraction; • Multi-scale representation with sortpooling	five-fold CV	HMDD v2.0/v3.2	0.939 (v2.0), 0.953 (v3.2)	https://github.com/cunmeiji/SGLMDA
**HGCLAMIR** (Ouyang et al., 2024) [33]	• HGCN; • Contrastive learning and view-aware attention mechanism; • Integrated multi-view representation	five-fold CV	HMDD v2.0/v3.2	0.9453 (v2.0), 0.9626 (v3.2)	https://github.com/OuyangDong/HGCLAMIR
**MHXGMDA** (Wen et al., 2024) [97]	• Multi-layer heterogeneous graph Transformer; • XGBoost classifier for decoding; • Multi-view similarity feature extraction	five-fold CV	HMDD v3.2	0.9594 (VG-data), 0.9601 (DA-data)	https://github.com/yinboliu-git/MHXGMDA
**HHMDA** (Dai et al., 2025) [89]	• Heterogeneous graph and hypergraph convolution; • Multi-scale feature extraction; • Laplacian regularization loss	five-fold CV	HMDD v3.2	0.9446 (RZ), 0.8713 (RC), 0.8692 (RM)	https://github.com/weiba/HHMDA
**MHMDA** (Li et al., 2025) [98]	• ”Similarity-association-similarity” metapath learning; • Heterogeneous-hyper network learning; • Multi-head attention aggregation	five-fold CV	HMDD v3.2	0.9557	https://github.com/ningq669/MHMDA
**SFPred** (Xuan et al., 2025) [83]	• Subgraph topology and dynamic graph topology enhanced graph learning; • Pairwise feature context association integration; • Multi-layer perceptron for dynamic topology	five-fold CV	HMDD v2.0	0.941	https://github.com/pingxuan-hlju/SFPred
**HeMDAP** (Ma et al., 2025) [99]	• Graph contrastive learning with meta-path view and network structure view; • Self-supervised and supervised contrastive learning; • Knowledge-aware enhancement	five-fold CV	HMDD v2.0/v3.2	0.9351 (v2.0), 0.9492 (v3.2)	https://github.com/IDATA-health/HeMDAP

“Unavailable” indicates that the “Source Code” is not provided in the corresponding paper. “AUC” is the area under the receiver operating characteristic curve. LOOCV AUC values in the table default to global LOOCV. “VG-data” and “DA-data” represent two miRNA–disease datasets. For detailed information, please refer to the MHXGMDA literature. “RZ” represents Random zeroing, “RC” represents Random multi-column zeroing, “RR” represents Random multi-row zeroing. “GL” represents global LOOCV.

#### Graph convolutional network

**Figure 10:**
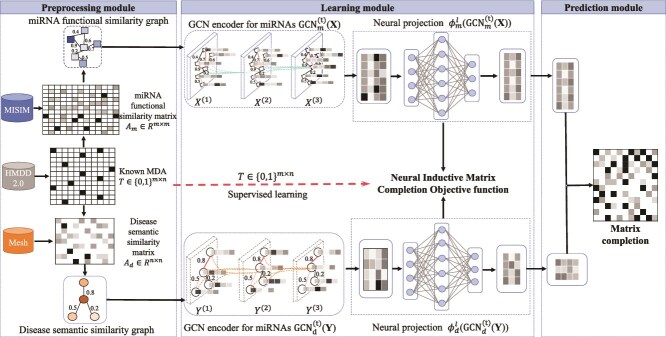
The framework of NIMCGCN model. $\mathbf{X}^{(1)}$ and $\mathbf{Y}^{(1)}$ are randomly initialized initial input miRNA and disease embeddings; $\mathbf{X}^{(2)}$ and $\mathbf{X}^{(3)}$ are the feature maps output by the second and third layer of the miRNA $\mathrm{GCN}_{\mathrm{m}}^{(2)}(\mathbf{X})$ and $\mathrm{GCN}_{\mathrm{m}}^{(3)}(\mathbf{X})$, while $\mathbf{Y}^{(2)}$ and $\mathbf{Y}^{(3)}$ are the feature maps from the second and third layer of the disease $\mathrm{GCN}_{\mathrm{d}}^{(2)}(\mathbf{Y})$ and $\mathrm{GCN}_{\mathrm{d}}^{(3)}(\mathbf{Y})$. $\phi _{m}^{l}(\cdot )$ and $\phi _{d}^{l}(\cdot )$ denote the total nonlinear transformations of the fully connected layers for miRNAs and diseases, respectively. The superscript $l$ indicates the $l$th layer of the fully connected network.

**I. Neural inductive matrix completion with GCNs (NIMCGCN)** Li *et al.* [81] proposed the NIMCGCN (see Fig. 10), which first uses GCN to learn latent feature representations of miRNAs and diseases from their respective similarity networks. These features are then fed into a novel neural IMC model to generate an association matrix completion. NIMCGCN is trained in a supervised end-to-end manner based on known MDAs.

**Figure 11:**
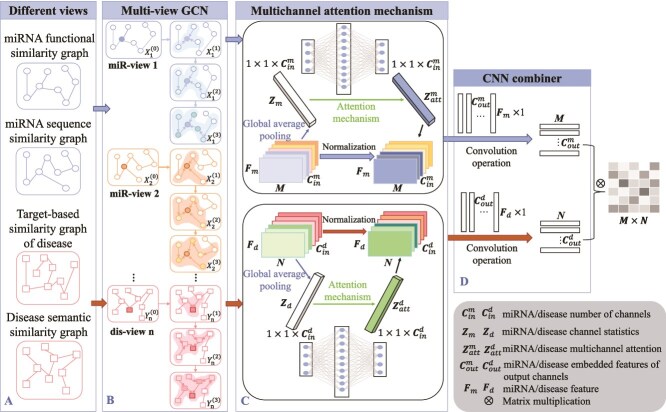
The framework of MMGCN model. (A) Multiple similarity graphs of miRNA and disease nodes are used as input. (B) Multi-view GCN encoder. The GCN encoder uses to fuse their neighbor information and generates miRNA and disease vectors from different perspectives. (C) The multichannel attention mechanism is divided into two sub-graphs: miRNA and disease. This mechanism focuses on the more important channel vectors and obtains normalized channel attention features. (D) The CNN combiner convolves the multichannel attention features of miRNA and disease to obtain their corresponding association prediction representations.

**II. Multi-view multichannel attention GCN for MDA Prediction (MMGCN)** Tang *et al.* [26] proposed MMGCN (see Fig. 11), which employs GCN to learn the feature representations of miRNAs and diseases multiple similarity perspectives. Moreover, MMGCN utilizes a multichannel attention mechanism to adaptively learn the importance of different features, focusing on more informative channels. Finally, a CNN combiner is used to fuse multi-channel features, generating unified embeddings for MDA prediction.

**III. Prediction of disease-related miRNAs based on multiple GCNs and random forest (MGCNRF)** Yang *et al.* [94] proposed MGCNRF, a model for predicting MDAs by integrating multiple GCNs and RF. The method first maps MFS, MSS, DSS, target similarity, and known MDAs into four different two-layer heterogeneous networks. Then, MGCNRF applies layered attention GCNs to extract embeddings of MDAs from different perspectives. Finally, these embeddings are integrated into the feature representation of miRNA–disease pairs, and potential associations are predicted using RF.

**IV. Hypergraph learning approach with improved GCN-Transformer for MDA prediction (HGTMDA)** Lu *et al.* [42] proposed HGTMDA, a hypergraph learning method that integrates a RWR association masking strategy and an enhanced GCN-Transformer model to address challenges in MDA prediction, such as data noise and insufficient integration of local and global information. The method begins by constructing homogeneous similarity networks for miRNAs and diseases and then adopts a novel association masking strategy that randomly obscures a subset of associations using RWR. This method, combined with attention-enhanced GCNs, effectively reduces noise and improves information extraction. Next, an miRNA–disease heterogeneous hypergraph is built, and an improved GCN-Transformer encoder is employed to capture both local structural features and global dependencies through multi-head attention and graph convolution operations. Finally, a combined dice cross-entropy (DCE) loss function is utilized to optimize model training, addressing class imbalance and probability distribution discrepancies.

**V. MDA prediction with multi-layer graph convolution and Negative Sample Selection (MGCNSS)** Tian *et al.* [82] proposed MGCNSS, which aims to address the limitations of existing methods in capturing meta-path associations and selecting reliable negative samples. MGCNSS first constructs a comprehensive heterogeneous network by integrating miRNA and disease similarity networks along with their known associations. It then employs multi-layer graph convolution to automatically capture meta-path associations with different lengths and learn discriminative representations of miRNAs and diseases. Additionally, MGCNSS selects high-quality negative samples using a distance-based strategy.

**VI. Subgraph topology and dynamic graph topology enhanced graph learning and pairwise feature context relationship integration for predicting MDAs (SFPred)** Xuan *et al.* [83] proposed SFPred, which first constructs neighborhood subgraphs for miRNA and disease nodes to capture local topological information. It then dynamically updates the edge weights between nodes using a MLP to form a dynamic graph topology. A convolutional Transformer module is designed to capture the context relationships among pairwise features, supplemented by a multi-perspective residual strategy to integrate detailed features.

#### Graph attention network

**I. Graph random propagation network and attention mechanism for MDA prediction (GRPAMDA)** Zhong *et al.* [91] proposed GRPAMDA, which combines a graph random propagation network based on DropFeature with an attention mechanism. The method first constructs a heterogeneous miRNA–disease graph using known MDA information. It then enhances node features via random propagation and aggregates the enhanced neighbor features using an attention mechanism. Finally, MDA scores are generated by a fully connected layer.

**II. Hierarchical GAT for MDA prediction (HGANMDA)** Li *et al.* [92] proposed HGANMDA, constructed a heterogeneous miRNA–disease-lncRNA graph and integrating node-layer attention and semantic-layer attention. The model projects miRNA and disease nodes into the same feature space, aggregates neighbor node features based on different meta-paths using node-layer attention, and learns the importance of these meta-paths via semantic-layer attention. The final node embeddings are obtained by fusing node aggregation features and semantic information. A bilinear decoder is then used to reconstruct MDAs.

**III. GATs and dual laplacian regularized least squares for MDA prediction (MKGAT)** Wang *et al.* [93] proposed MKGAT, a computational method for predicting MDAs using graph attention network (GAT) and dual Laplacian RLS. MKGAT extracts features from known MDAs, MFS, and DSS using GAT. It then calculates GIPK for multi-layer embeddings and fuses these kernels with initial similarities using an attention mechanism. Finally, dual Laplacian RLS are applied for association prediction.

#### Graph autoencoder

Li *et al.* [84] proposed GAEMDA, which integrates a GAE and a bilinear decoder to generate embeddings of miRNAs and diseases in an end-to-end manner. The method projects heterogeneous miRNA and disease features into the same vector space and aggregates neighborhood information using GNNs to enhance feature representation. The embeddings are then fed into a bilinear decoder to reconstruct MDAs. Ding *et al.* [85] proposed the VGAMF to predict MDAs by integrating multi-view data sources (e.g. MSS, MFS, and DSS) into comprehensive similarity networks. The method employs variational GAE to extract nonlinear representations of miRNAs and diseases from these networks, while NMF is used to obtain linear representations based on the known MDA matrix. Finally, a fully connected neural network combines these linear and nonlinear representations to generate predicted association scores for all miRNA–disease pairs. Zhang *et al.* [86] proposed AGAEMD, which uses a node-level attention GAE. The method employs a node-level attention-based GAE to learn low-dimensional dense embeddings for nodes. The embeddings from multiple layers are aggregated using a jumping knowledge module, and the final association scores are reconstructed via an inner product decoder.

#### Hypergraph neural network

**I. Attention aware multi-view similarity networks and hypergraph learning for MDAs identification (AMHMDA)** Ning *et al.* [87] proposed AMHMDA, which first constructs multi-view similarity networks for miRNAs and diseases, and extracts useful similarity information using GCN combined with attention mechanisms. To obtain high-quality links and richer node information, hypernodes are introduced to construct a heterogeneous hypergraph of miRNAs and diseases. Finally, layer-level attention is employed to fuse the outputs of GCN layers, and the association scores are predicted using fully connected layers.

**Figure 12:**
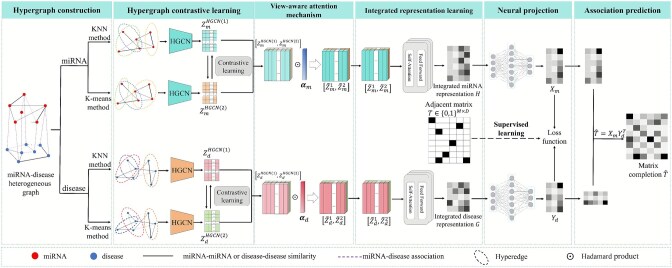
The framework of HGCLAMIR model. In the formula, $\boldsymbol{Z}^{HGCN(1)}_{m}$ and $\boldsymbol{Z}^{HGCN(2)}_{m}$ represent the miRNA embeddings of the first and second views output by the HGCN model, respectively. $\boldsymbol{a}_{m}$ denotes the attention weight. $\boldsymbol{\tilde{Z}}^{1}_{m}$ and $\boldsymbol{\tilde{Z}}^{2}_{m}$ are obtained by combining the embedding representations of different views with attention weights. For diseases, the calculation symbols are exactly the same as those for miRNAs, except that the subscript is changed to “$d$” for representation. $ X_{m} $ and $ Y_{d} $ denote the final miRNA and disease embedding representation matrix.

**II. Hypergraph contrastive learning with attention mechanism and integrated multi-view pepresentation for MDA prediction (HGCLAMIR)** Ouyang *et al.* [33] proposed HGCLAMIR (see Fig. 12), a computational method for predicting MDAs by integrating HGCNs, contrastive learning, view-aware attention mechanisms, and integrated multi-view representations. HGCLAMIR captures high-order complex associations using HGCN and enhances embedding representation learning through contrastive learning. The model further improves prediction performance by adaptively weighting different views with a view-aware attention mechanism and integrating multi-view information.

**Figure 13:**
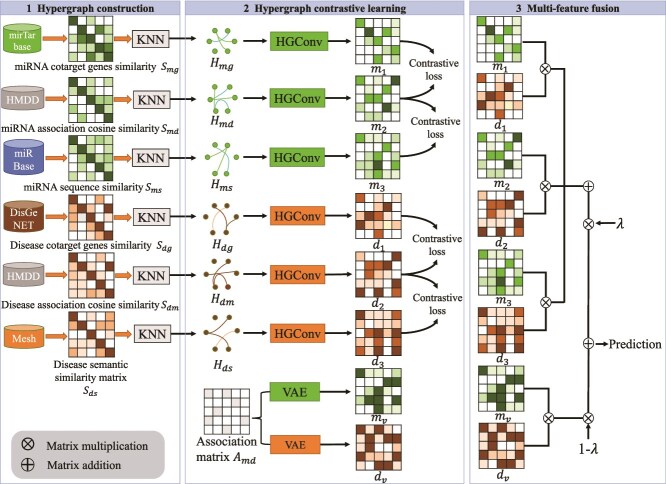
The framework of MHCLMDA model. Hypergraph ${H}_{mg}$ is constructed based on the similarity of cotarget genes, hypergraph ${H}_{md}$ is built based on the cosine similarity of miRNAs in disease associations, and hypergraph ${H}_{ms}$ is established based on the second-order sequence similarity of miRNAs. ${H}_{dg}$ is derived from the similarity of disease co-associated genes, ${H}_{dm}$ is based on the cosine similarity of diseases in miRNA associations, and ${H}_{ds}$ is based on the second-order semantic similarity of diseases. $m_{1}$ represents the miRNA feature derived from ${H}_{mg}$, $m_{2}$ refers to the miRNA feature obtained from ${H}_{md}$, $m_{3}$ denotes the miRNA feature extracted from ${H}_{ms}$. $d_{1}$ denotes the disease feature extracted from ${H}_dg$, $d_{2}$ represents the disease feature obtained from ${H}_{dm}$, $d_{3}$ refers to the disease feature derived from ${H}_{ds}$. $m_{v}$ denotes the final miRNA key features, $d_{v}$ denotes the final disease key features.

**III. Multihypergraph contrastive learning for MDA prediction (MHCLMDA)** Peng *et al.* [88] proposed MHCLMDA (see Fig. 13), whose core methodology involves constructing multiple miRNA and disease hypergraphs based on diverse data sources (e.g. MSS, DSS, miRNA target genes, and disease-associated genes). Hypergraph convolution is applied to each hypergraph to capture high-order associations among nodes, while contrastive learning ensures consistent feature representations across different views. A variational auto-encoder (VAE) is employed to extract nonlinear key features from known MDAs. Finally, MHCLMDA fuses multi-view features for association prediction, with model parameters optimized end-to-end.

**IV. Heterogeneous hypergraph convolution and heterogeneous graph multi-scale convolution (HHMDA)** Dai *et al.* [89] proposed HHMDA, which first constructs a heterogeneous miRNA–disease graph and captures multi-scale feature representations of miRNAs and diseases using graph convolution. These features are then used to reconstruct the MDA matrix. Meanwhile, HHMDA builds a heterogeneous hypergraph with miRNAs and diseases as nodes, where hyperedges consist of miRNAs and diseases linked to the same genes. Hypergraph convolution is performed to extract high-order features, which are combined with Laplacian regularization loss to optimize the model.

#### Others

**I. Meta-path-based MDA prediction (MDPBMP)** Yu *et al.* [90] proposed MDPBMP, which addresses the limitations of insufficient meta-path information and potential association exploration in existing methods. The method constructs a heterogeneous miRNA–disease information network and defines seven symmetrical meta-paths to extract node feature information. By aggregating information from all nodes on meta-path instances and using multi-head attention mechanisms, MDPBMP generates embedding feature vectors for miRNAs and diseases to calculate association scores.

**II. Hybrid high-order moment residual model for MDA prediction (HHOMR)** Li *et al.* [95] proposed HHOMR, which constructs a heterogeneous graph based on HMDD v2.0, combining MFS, DSS, and GIPK similarity to generate node feature matrices. HHOMR employs a structural fusion layer to capture graph topology and a hybrid high-order moment encoder layer to enhance node feature distribution. An element-level attention mechanism adaptively integrates multi-order moment features, and a multi-layer perceptron computes association scores.

**III. SubGraph learning-based method for MDA prediction (SGLMDA)** Ji *et al.* [96] proposed SGLMDA, which integrates multi-view of similarities (including DSS, MFS, MSS, and GIPK similarity) to construct a heterogeneous graph. For each miRNA–disease pair, SGLMDA samples *K*-hop subgraphs and employs a GNN-based algorithm to extract subgraph representations for prediction.

**Figure 14:**
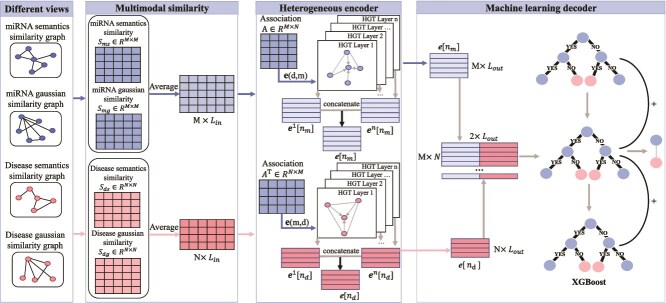
The framework of MHXGMDA model. In the formula, **e**(d,m) denotes the edge from the source node (disease $d$) to the target node (miRNA $m$), which is a specific type of association edge connecting diseases and miRNAs in the heterogeneous graph. Similarly, **e**(m,d) represents the edge from miRNA $m$ to disease $d$. $\mathbf{\textit{e}}^{1}[n_{m}]$ and $\mathbf{\textit{e}}^{1}[n_{d}]$ represent the updated embedding vector of the miRNA node $n_{m}$ and disease node $n_{d}$ after being processed by the first layer of the multilayer HGT. $\mathbf{\textit{e}}^{n}[n_{m}]$ and $\mathbf{\textit{e}}^{n}[n_{d}]$ represent the updated embedding vectors of miRNA node $n_{m}$ and disease node $n_{d}$ after being processed by the $n$th layer of HGT. $\mathbf{\textit{e}}[n_{m}]$ and $\mathbf{\textit{e}}[n_{d}]$ denote the miRNA and disease final embedding obtained by concatenating the node embedding vectors from the first to the $n$th layer.

**IV. Multilayer heterogeneous graph transformer and XGBoost for MDA prediction (MHXGMDA)** Liu *et al.* [97] proposed MHXGMDA (see Fig. 14), which uses multi-view similarity matrices (including MSS, DSS, and GIPK similarity) to construct biological feature vectors. The multi-layer heterogeneous graph Transformer captures dynamic and high-order associations between miRNAs and diseases, while the XGBoost classifier decodes the embedded features to predict association scores.

**V. Similarity-association-similarity metapaths and heterogeneous-hyper network learning (MHMDA)** Li *et al.* [98] proposed MHMDA to address the limitations of insufficient long-distance pathway information and potential association exploration. The core innovation lies in the integration of hierarchical attention-based multi-hop metapaths and a heterogeneous-hyper network (HeteroHyperNet). MHMDA constructs multi-view similarity networks using MSS, MFS, and DSS, followed by learning long-distance associations through “similarity-association-similarity” metapaths with hierarchical attention. The HeteroHyperNet combines heterogeneous graph and hypergraph learning via progressive aggregation, capturing both known and potential global associations. Multi-head attention aggregates features from metapaths and HeteroHyperNet, generating final prediction scores.

**VI. Heterogeneous graph self-supervised learning for MDA prediction (HeMDAP)** Ma *et al.* [99] proposed HeMDAP, a method for MDA prediction that integrates network structure and meta-path views of heterogeneous graphs. The model leverages self-supervised and supervised contrastive learning to optimize node embeddings and incorporates knowledge-aware enhancement to improve embedd-ing quality. This multi-view, multi-task learning method comprehensively captures complex associations among miRNAs, genes, and diseases.

## Experimental results

### Data preparation

To ensure fairer comparison and reproducibility between different computational methods, and to ensure that model comparisons only reflect differences in algorithmic performance rather than accidental biases caused by data preprocessing, we used the standardized dataset established by MMGCN (https://github.com/Txinru/MMGCN) [26], which is based on HMDD v3.2 [100]. The original HMDD v3.2 dataset contains 35 547 experimentally validated MDAs involving 1206 miRNAs and 894 diseases. After data cleaning, including deduplication and removal of miRNAs lacking sequence annotations, the resulting dataset contained 12 446 high-confidence associations covering 853 miRNAs and 591 diseases.

To address the data leakage issue highlighted in previous research [101], we strictly excluded any GIPK similarities directly computed from the MDA matrix in all comparative experiments. It is important to note that introducing GIPK similarities before performing cross-validation can cause test-set positive samples to be implicitly used during model training, thereby resulting in data leakage and compromising the reliability of the experimental evaluation. Therefore, this paper uses the following four similarity networks to construct multi-view networks: MFS, MSS, DSS, and DFS. For the fairness and consistency of the comparative evaluation, the GIPK similarities used in the 11 baseline models were uniformly replaced with alternative similarity networks, ensuring that all models were compared on the same dataset.

Specifically, in SLMRWMDA, IMCMDA, PCACFMDA, MvKFN-MDA, NIMCGCN, HGCLAMIR, and MHXGMDA, the original miRNA and disease GIPK similarities were replaced with MSS and DFS, respectively. For the MLRDFM model, which originally incorporates MFS, MSS, DSS, miRNA GIP kernel similarity, miRNA semantic similarity, and disease GIP kernel similarity, additional adjustments were necessary to ensure that all baseline models were evaluated under the same data setting. In this case, the miRNA GIPK similarity and miRNA semantic similarity were removed, and the disease GIPK similarity was substituted with DFS. For the SPLHRNMTF model, which originally used MFS, MSS, miRNA GIPK similarity, DSS, and disease GIPK similarity, we removed the miRNA GIPK similarity and replaced the disease GIPK similarity with DFS. For the MLRDFM model that originally included MFS, MSS, DSS, miRNA GIPK similarity, miRNA semantic similarity, and disease GIPK similarity, we removed miRNA GIPK similarity and miRNA semantic similarity, and replaced disease GIPK similarity with DFS. Similarly, the MHCLMDA model originally employed miRNA cotarget-gene similarity, miRNA association cosine similarity, miRNA sequence similarity, disease cotarget-gene similarity, and disease association cosine similarity. We used only MFS, MSS, DSS, and DFS, and removed all other similarity.

### Experimental design and evaluation metrics

To evaluate the performance of all models, we employed five-fold CV with random sampling, dividing the dataset into five equal subsets, four for training and one for testing. The known MDAs were treated as positive samples and randomly partitioned into five disjoint subsets. To ensure a rigorous evaluation, all datasets were carefully filtered to prevent any overlap between training and test sets. When conducting comparative experiments, all models used the best hyperparameters from the original studies. Model performance was assessed using seven metrics: AUC, the area under the precision-recall curve (AUPR), F1-score, accuracy, precision, recall, and specificity.

### Comparative experiments

To ensure the fairness and comprehensive comparison, two selection criteria are adopted in the paper: (i) one to three representative or top-performing models in each category (NW-based, MF-based, ML-based, DL-based, and GNN-based methods) is/are selected; and (ii) models with open source availabilities are chosen to ensure unbiased reproducibility. Based on these criteria, we have identified 11 computational methods for rigorous comparison. We conducted comparative experiments on the dataset using the optimal parameter reported in each paper. The results are listed in Table 7.

**Table 7:** Performance comparison on HMDD v3.2

Category	Model	AUC	AUPR	F1	Accuracy	Recall	Specificity	Precision
NW-based	SLMRWMDA [49]	0.9233444	0.9311169	0.8613080	0.8592206	0.8740860	0.8443552	0.8492021
MF-based	IMCMDA [56]	0.8195813	0.8175556	0.7598793	0.7335207	0.8433613	0.6236802	0.6919014
MF-based	SPLHRNMTF [53]	0.9289440	0.9336144	0.8577363	0.8543220	0.8782671	0.8303768	0.8384196
ML-based	PCACFMDA [61]	0.9842628	0.9844457	0.9446782	0.9447096	0.9420905	0.9473756	0.9472985
DL-based	MvKFN-MDA [77]	0.9275070	0.9356222	0.8663325	0.8639172	0.8822820	0.8455524	0.8513374
DL-based	MLRDFM [78]	0.9405450	0.9403899	0.8687587	0.8650572	0.8932255	0.8370648	0.8459003
GNN-based	NIMCGCN [81]	0.9216308	0.9313624	0.8637881	0.8627039	0.8706469	0.8547610	0.8573467
GNN-based	MMGCN [26]	0.9277104	0.9358860	0.8673959	0.8659662	0.8766894	0.8552430	0.8586448
GNN-based	MHCLMDA [88]	0.9254666	0.9349591	0.8598935	0.8600346	0.8588293	0.8612399	0.8610570
GNN-based	HGCLAMIR [33]	0.9507844	0.9480827	0.8834353	0.8804902	0.9057614	0.8552190	0.8625228
GNN-based	MHXGMDA [97]	0.9574839	0.9523965	0.8920726	0.8865006	0.9392598	0.8338684	0.8493998

**Method Categories:** NW-based (network-based methods), MF-based (matrix factorization-based methods), ML-based (machine learning-based methods), DL-based (non-graph deep learning-based methods), GNN-based (graph neural network-based methods).

The experimental results demonstrate that GNN-based methods outperform most NW-based, MF-based, ML-based, and DL-based methods across multiple evaluation metrics, including AUC, AUPR, F1-score, accuracy, recall, specificity, and precision. Specifically, NW-based and MF-based methods generally exhibit relatively weaker performance across most metrics. For example, the MF-based IMCMDA model achieves an AUC of only 0.8196. This limitation primarily stems from the heavy reliance of these methods on manually constructed similarity networks or low-rank matrix factorization assumptions, which often fail to effectively capture the nonlinear and deep-level latent information underlying MDAs.

ML-based methods typically achieve better performance by integrating handcrafted features with flexible classifiers, but their dependence on feature engineering restricts their ability to fully model complex heterogeneous network structures. In contrast, DL-based methods can learn nonlinear representations in an end-to-end manner, leading to improved performance across multiple metrics. For instance, MvKFN-MDA employs fully connected neural networks to learn high-quality representations of miRNAs and diseases, achieving an AUC of 0.9275. However, many DL-based approaches do not fully exploit the inherent topological priors in biological networks, which limits their ability to capture relational information.

Among all categories, GNN-based methods show the most overall superior performance, exhibiting higher accuracy and robustness. This advantage arises from their ability to directly model graph-structured data, capture multi-hop dependencies, and integrate heterogeneous biological features within an end-to-end framework. Nevertheless, the performance of GNN-based methods remains highly dependent on the quality of the graph construction and the design of the model architecture. Poorly constructed graph structures or inadequately designed message-passing mechanisms may result in performance that is even lower than that of ML-based methods with carefully engineered features and robust training strategies. In particular, the dataset used in our comparative experiments is extremely sparse: it contains 12 446 high-confidence MDAs covering 853 miRNAs and 591 diseases, whereas the number of unknown potential associations reaches 491 677, yielding an edge density of 2.5%. Such sparsity severely limits the availability of informative neighbors, making it difficult for GNN-based methods to fully leverage neighborhood structures to learn effective representations. More importantly, in the current comparative experiments, only two out of five GNN-based methods outperform ML-based methods. Notably, the ML-based PCACFMDA model achieves an AUC of 0.9843, significantly exceeding that of the GNN-based MHXGMDA model, which has an AUC of 0.9575. This observation supports the aforementioned point. The superior performance of PCACFMDA may be attributed to its hybrid architecture, where PCA-based dimensionality reduction combined with a cascade forest ensemble generates high-quality low-dimensional representations, effectively reducing noise and enhancing feature discriminability. Therefore, GNN-based methods still require careful design in graph construction, information propagation mechanisms, and feature integration strategies to fully leverage their potential in complex biological networks and achieve more competitive predictive performance.

To evaluate the generalizability of each baseline model in predicting new MDAs, we conducted global leave-one-out cross-validation (global LOOCV) experiments and plotted the ROC curves for all methods (see Supplementary Figure S1). The results indicate that the AUC performance of different models under global LOOCV exhibits some fluctuation compared with five-fold CV, suggesting that strong performance in five-fold CV does not necessarily reflect a model’s ability to predict entirely new associations. Therefore, the generalization capability to new miRNA and disease associations should be carefully considered when designing prediction models.

## Discussions and future directions

### Discussions

The performance of the computational methods continue to improve in the last decade (the AUC increased from 0.845 to 0.987). It may be due to three technologies: algorithm fusion innovations transitioning from shallow networks (PBMDA) to combining PCA dimensionality reduction and cascade forest (PCACFMDA); enhanced data utilization through expanded HMDD datasets (from version 2.0 to version 3.2); and other technical support like contrastive learning in MHCLMDA and Laplacian constraints in HHMDA.

Computational methods for MDA prediction have their own advantages and disadvantages and need to be carefully selected based on specific research goals and data availability. The NW-based methods, such as RWR, can integrate multi-omics networks including miRNA–disease, miRNA–gene, and disease–gene to infer potential associations. Although powerful in exploiting topological associations, their performance relies heavily on the completeness of known MDAs. Sparse networks can lead to unreliable propagation, especially for new miRNAs or diseases with cold start problems.

The MF-based methods provide flexibility for fusing heterogeneous data through latent space projection. However, linear decomposition has difficulty in simulating nonlinear biological interactions and extracting deep features. The ML-based methods can effectively predict new miRNA–disease pairs, but they perform poorly in capturing complex deep features. The linear assumption of these methods makes it difficult to capture nonlinear information in MDA networks in sparse or heterogeneous datasets. The DL-based methods address the limitations of MF-based methods by extracting nonlinear representations through deep features. However, their black-box nature hinders biological interpretability. The GNN-based methods are good at aggregating topological and attribute information, among which hypergraph show better performance. However, the cold start problem remains to be solved because the neighborhood information of new miRNAs or disease nodes is insufficient for effective information transfer.

In recent years, research related to MDA prediction has expanded from the single task of MDA inference to the broader field of ncRNA biomarker discovery. For example, in circRNA–miRNA interaction prediction, several methods have incorporated multisource information and deep feature extraction strategies, such as DeepCMI [102], KGDCMI [103], and JSNDCMI [104]. In the study of lncRNA–miRNA–disease associations, multiple reviews have summarized recent advances in computational prediction methods [105, 106]. Moreover, increasingly comprehensive ncRNA-disease prediction frameworks have been developed, including ceRNA–disease association models [107] and methods designed to identify previously unknown cancer biomarkers [108, 109]. Collectively, these studies demonstrate that diverse types of ncRNAs are connected through intricate and multilayered regulatory relationships. Such complex interactions are crucial for understanding disease mechanisms and for improving computational models of ncRNA–disease association prediction.

This review investigated 66 computational methods, with DL-based methods and GNN-based methods achieving better performance, especially when integrating multi-view biological networks into the models. However, it should be noted that this study has two main limitations: (i) the comparative experiments were conducted only on a single standardized dataset, HMDD v3.2. Although this dataset has been widely adopted, the generalizability of the comparative experimental results to datasets from different sources or different updated versions still needs further verification; (ii) the hyperparameters of all baseline models are directly set according to the original literature and have not been fine-tuned for this dataset. They may not be optimal, which may have a potential impact on performance comparison.

### Future directions

Despite significant advancements in computational methods for MDA prediction, several critical challenges remain unresolved. We outline five key research directions to advance the field.

**I. Interpretable graph representation learning.** While GNN-based methods achieve better prediction performance, their “black box” nature limits biological interpretability. Future models should integrate interpretability methods, including attention mechanisms [33] or meta-path [110, 111], to reveal regulatory pathways between miRNAs and diseases.

**II. Cold start problem.** In MDA prediction, the model cannot accurately predict the potential relationship of newly emerged miRNAs or diseases due to the lack of known association data. In the future, zero-shot learning or few-shot learning strategies could be incorporated to address this limitation [112]. In addition, IMC methods inherently prioritize miRNAs or diseases without known MDAs, thereby improving the model’s adaptability to cold-start scenarios involving novel miRNAs or diseases [56].

**III. Multi-view fusion strategy.** The outstanding performance of multi-view models such as MMGCN and AMHMDA highlights the importance of heterogeneous data integration. Future research could consider dynamically weighting different views during model design, based on their importance to the prediction results, such as MFS, MSS, DFS, and DSS. This could also further consider shared and complementary information between views [33].

**IV. Optimize negative sampling mechanisms.** The common practice of random negative sampling introduces false negatives, which reduces the reliability of the model, especially for newly discovered miRNAs or diseases. Future methods can leverage Positive and Unlabeled Learning (PU Learning) algorithms [113] to achieve confidence-weighted sampling, assigning lower weights to potential positive samples in unlabeled data. Moreover, the WKNKN algorithm [114] can be incorporated to further refine the negative sampling mechanisms. To alleviate the issue that random negative sampling may introduce potential false negatives, we further incorporated a WKNKN-based negative sample correction strategy and a reliable negative sample selection strategy based on PU Learning. The experimental results in Supplementary Tables S1 and S2 show that both types of methods improve the predictive performance of the model to varying degrees compared with random negative sampling. This demonstrates that designing more appropriate negative sampling mechanisms can indeed effectively mitigate the adverse impact of false negatives and other noisy labels on model training.

**V. miRNA dysregulation type-disease association prediction.** Existing models can predict binary MDAs, but few studies have predicted the association between disease and up-regulation/down-regulation of miRNA. Yu *et al.* [115] proposed the MRFGMDA algorithm to predict the association between up-regulated, down-regulated or dysregulated miRNA and disease. In future studies, the signed GNN [116] can be used to expand the computational method to predict the association between disease and up-regulated or down-regulated miRNA. This method requires a carefully selected dataset that contains annotations of the type of disorder, and data cleaning is required to ensure that each miRNA has at least one up-regulated and one down-regulated disease-related.

Combining these future directions with emerging single-cell multi-omics data is essential for developing clinically actionable models. In addition, truly combining computational methods with wet experiments is essential for verifying the predicted mechanisms of computational methods and promoting innovation in disease treatment, thereby achieving the goal of using computational methods to improve the efficiency of biological experiments and save time and costs.

## Conclusion

The understanding of miRNAs evolves significantly over time since their initial identification in 1993 in *Caenorhabditis elegans*. Dysregulated expression of miRNA may lead to the occurrence and development of a variety of human diseases [117]. miRNAs are considered potential disease biomarkers, especially in the field of cancer. Predicting unknown MDA is crucial for the diagnosis, treatment, and prevention of diseases. Computational methods for MDA prediction can serve as a preliminary screening tool over the traditional wet-lab experiments. Accelerating the discovery of new MDAs saves cost and time. In this article, we comprehensively review different computational methods for MDA prediction, and conduct multidimensional analysis of miRNAs. First, we briefly introduce the biological mechanisms and development history of miRNAs and analyze data resources related to MDA and similarity matrix calculation methods. Second, five computational methods (network-, matrix factorization-, machine learning-, non-graph deep learning-, and GNN-based) for MDA prediction are elaborated. Third, different types of computational methods for comparative experiments and research trends are discussed. Finally, we state potential future research directions.

**Key Points**

- Different miRNA functions and their respective biogenesis mechanisms will be discussed. A review of the discovery history and research development of miRNAs will be outlined.
- We systematically summarize different relevant MDA databases and introduce various miRNA and disease similarity calculation methods.
- Existing computational methods for predicting MDAs are comprehensively classified into five major categories. Among them, 11 representative approaches are selected for performance comparison through experiments. Through the results, we can provide an in-depth analysis of their strengths, limitations, and future challenges.
- A GitHub repository is established and maintained for hosting curated database information, tracking and continuously updating the latest developments in the five identified computational categories.

## Supplementary Material

Supplementary_Figure_S1_bbaf736

Supplementary_Table_S1_bbaf736

Supplementary_Table_S2_bbaf736

Supplementary_Text_1_bbaf736

Supplementary_Text_2_bbaf736

Supplementary_Text_3_bbaf736

Supplementary_Text_4_bbaf736

## Data Availability

The datasets for the comparative experiments in this review are available at https://github.com/xiesiya/miRNA-disease-association-methods.
