# Supplementary figures and images for: From biogenesis to deep modeling: a holistic review of miRNA–disease prediction computational methods with experimental comparison
Source: Brief Bioinform. 2026 Jan 19;27(1):bbaf736. doi: 10.1093/bib/bbaf736 (PMC12814990; doi:10.1093/bib/bbaf736)

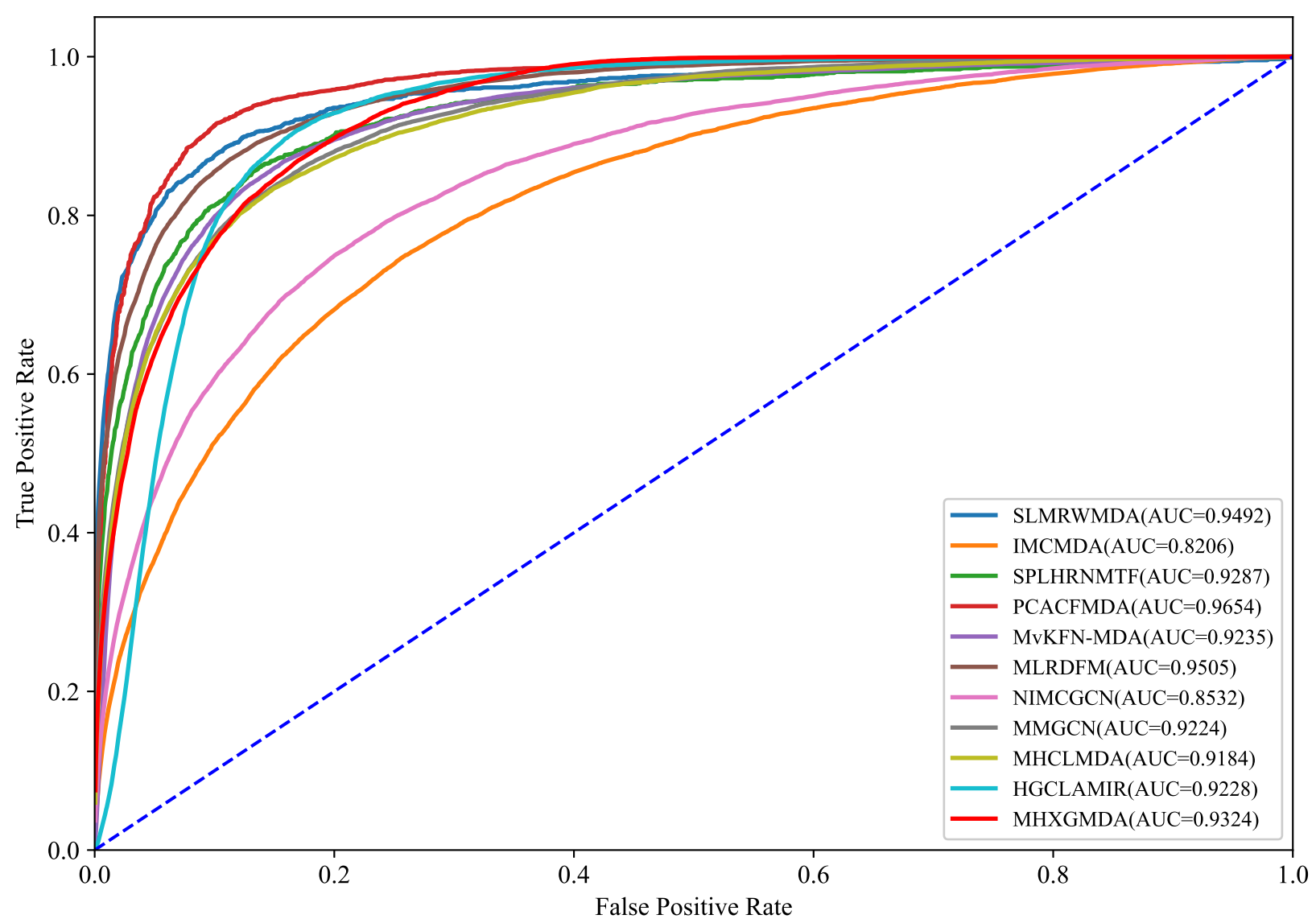

Supplement: Supplementary_Figure_S1_bbaf736 [file supplementary_figure_s1_bbaf736.pdf]
