# Supplementary material for: From biogenesis to deep modeling: a holistic review of miRNA–disease prediction computational methods with experimental comparison
Source: Brief Bioinform. 2026 Jan 19;27(1):bbaf736. doi: 10.1093/bib/bbaf736 (PMC12814990; doi:10.1093/bib/bbaf736)
Supplement: Supplementary_Text_1_bbaf736 [file supplementary_text_1_bbaf736.pdf]

Supplementary Text 1

Overview of ncRNAs

There are various RNA molecules within cells, which can be divided into two major categories based on whether they carry protein-coding information: mRNA and ncRNA. Protein-coding genes serve as repositories of genetic information in DNA, while mRNA functions as the intermediary carrier that conveys this information for protein synthesis in the cytoplasm. Transcribed from these protein-coding genes and subsequently processed, mRNA harbors the precise instructions required to synthesize proteins. In contrast, ncRNAs, despite not encoding proteins, are critical regulators of gene expression and overall cellular function. They are involved in a range of biological processes, including organismal development, tissue differentiation, and cell cycle regulation [1]. Based on transcript length, ncRNAs can be further classified into small ncRNAs and long ncRNAs, as shown in Table 1. Long ncRNAs is defined as RNA molecules longer than 200 nt [2].

Table 1: ncRNA classification based on transcript length

ncRNA Type	Subtype	Length	Functional Description
**long ncRNAs**	lncRNA	> 200 nt	Regulation at transcriptional and post-transcriptional levels
	circRNA	> 200 nt	Circular; regulates gene expression and acts as miRNA "sponge"
	Xist RNA	> 200 nt	Involved in X-chromosome inactivation and sex determination in female mammals
**small ncRNAs**	miRNA	21–25 nt	Post-transcriptional regulation of gene expression
	siRNA	20–25 nt	RNA interference and mRNA silencing
	piRNA	24–31 nt	Protects genome integrity in germ cells from transposons
	snoRNA	< 200 nt	Modifies and processes rRNA, tRNA, and snRNA
	tRNA	< 200 nt	May regulate protein synthesis
	rRNA	< 200 nt	Structural and functional core of ribosomes; involved in translation
	snRNA	< 200 nt	Forms spliceosomes with proteins; involved in pre-mRNA splicing

Major types of long ncRNAs include lncRNAs, circular RNAs (circRNAs), and Xist RNA, the latter of which is involved in the process of X-chromosome inactivation. Small ncRNAs typically have lengths of less than 200 nt and include microRNA (miRNA), small interfering RNA (siRNA), PIWI-interacting RNA (piRNA), small nucleolar RNA (snoRNA), transfer RNA (tRNA), ribosomal RNA (rRNA), and small nuclear RNA (snRNA).

Among small ncRNAs, miRNAs stand out as one of the most functionally significant classes. miRNA is an endogenous single-stranded ncRNA molecule that plays a key regulatory role in gene expression. By binding to complementary sequences of target mRNAs, miRNAs either degrade their targets or suppress translation, thereby fine-tuning protein synthesis with remarkable precision. This post-transcriptional regulatory mechanism is predicted to control the activity of approximately 30% of all protein-coding genes in mammals, and miRNAs have been shown to participate in the regulation of nearly every cellular process investigated so far [3].

References

[1] Lu, J., Getz, G., Miska, E.A., Alvarez-Saavedra, E., Lamb, J., Peck, D., Sweet-Cordero, A., Ebert, B.L., Mak, R.H., Ferrando, A.A., *et al.*: Microrna expression profiles classify human cancers. nature **435**(7043), 834–838 (2005)

[2] Nagano, T., Fraser, P.: No-nonsense functions for long noncoding rnas. Cell **145**(2), 178–181 (2011)

[3] Filipowicz, W., Bhattacharyya, S.N., Sonenberg, N.: Mechanisms of post-transcriptional regulation by micrornas: are the answers in sight? Nature reviews genetics **9**(2), 102–114 (2008)
