# Supplementary material for: From biogenesis to deep modeling: a holistic review of miRNA–disease prediction computational methods with experimental comparison
Source: Brief Bioinform. 2026 Jan 19;27(1):bbaf736. doi: 10.1093/bib/bbaf736 (PMC12814990; doi:10.1093/bib/bbaf736)
Supplement: Supplementary_Text_2_bbaf736 [file supplementary_text_2_bbaf736.pdf]

Supplementary Text 2

Analyzing the generation mechanism of miRNA

The generation of miRNAs is a highly coordinated, multi-step process that begins in the nucleus and culminates in the cytoplasm. As shown in figure 1, miRNAs are initially transcribed as long primary miRNAs (`Pri-miRNAs`) by `RNA polymerase II` or `III` (`RNA Pol II/III`). These `Pri-miRNAs` are processed within the nucleus by the `Drosha` and `Pasha` enzyme, which cleaves them into precursor miRNAs (`Pre-miRNAs`) of approximately 70 nt in length. The `Pre-miRNAs` are then transported to the cytoplasm via `Exportin-5`, where the `Dicer` enzyme further processes them into mature miRNA duplexes of 20-25 nt. One strand of the duplex is loaded into the RNA-induced silencing complex (RISC), while the other strand is degraded. The mature miRNA within RISC then directs the complex to target mRNAs, where it either facilitates mRNA cleavage or inhibits translation, depending on the degree of complementarity between the miRNA and its target [1].

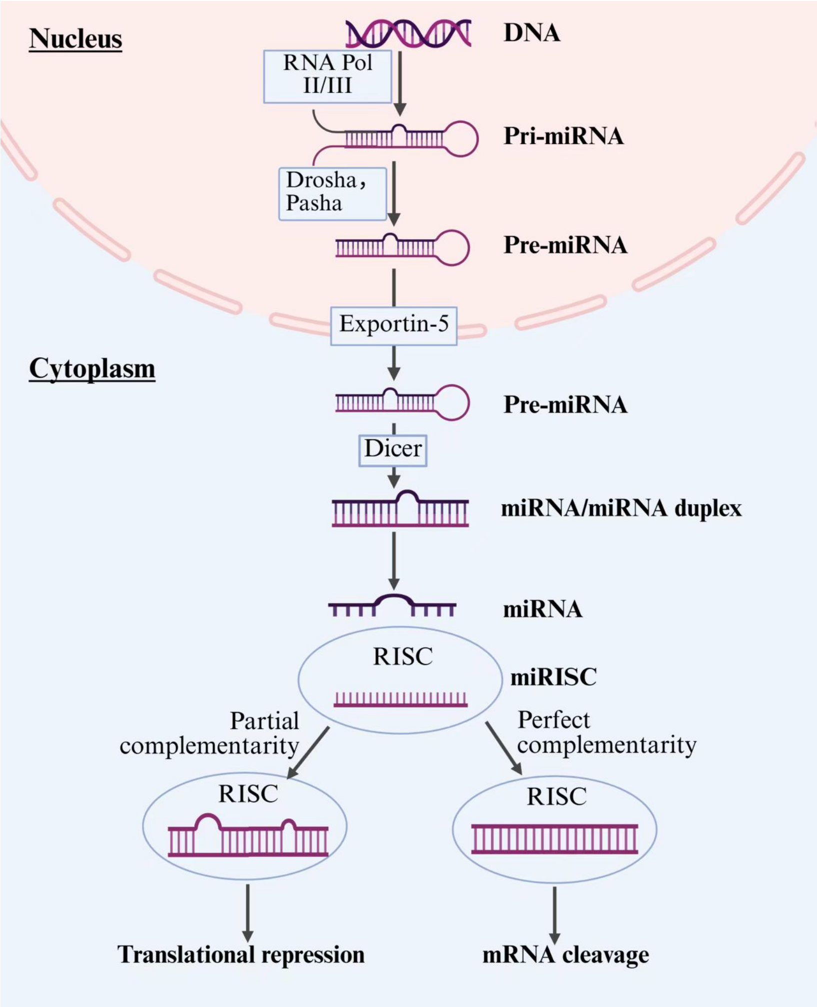

Fig. 1: The biosynthesis of miRNAs and their function in the cytoplasm.

As key post-transcriptional regulators, miRNAs regulate gene expression by complementary base pairing with target mRNAs. While miRNAs are best known for binding to the mRNAs 3'UTR, they can also interact with the 5' untranslated region (5'UTR) or the coding region, leading to either mRNA cleavage or translation inhibition [2]. miRNAs have a dual mechanism of action, namely degradation or translational repression, which enables them to have a profound impact on cellular processes such as cell proliferation, differentiation, apoptosis, and immune responses.

Beyond their direct effects on mRNA stability and translation, miRNAs also influence gene expression indirectly by regulating transcription factors that bind to promoter regions [3]. This adds another layer of complexity to their regulatory roles, as they can modulate the transcriptional activity of genes in addition to their post-transcriptional effects. Furthermore, miRNAs are embedded in intricate regulatory networks involving other ncRNAs, such as lncRNAs and circRNAs. These lncRNAs and circRNAs can act as molecular sponges or decoys, sequestering miRNAs and modulating their availability within the cell [4, 5]. This dynamic interplay between miRNAs and other ncRNAs underscores the central role of miRNAs in maintaining cellular homeostasis and highlights their potential as therapeutic targets in disease contexts.

References

[1] Zeng, Y., Yi, R., Cullen, B.R.: Micrornas and small interfering rnas can inhibit mrna expression by similar mechanisms. Proceedings of the National Academy of Sciences **100**(17), 9779–9784 (2003)

[2] Bartel, D.P.: Micrornas: genomics, biogenesis, mechanism, and function. cell **116**(2), 281–297 (2004)

[3] Hobert, O.: Gene regulation by transcription factors and micrornas. Science **319**(5871), 1785–1786 (2008)

[4] Ma, B., Wang, S., Wu, W., Shan, P., Chen, Y., Meng, J., Xing, L., Yun, J., Hao, L., Wang, X., *et al.*: Mechanisms of circrna/lncrna-mirna interactions and applications in disease and drug research. Biomedicine & Pharmacotherapy **162**, 114672 (2023)

[5] Zhang, Y., Zhan, L., Jiang, X., Tang, X.: Comprehensive review for non-coding rnas: From mechanisms to therapeutic applications. Biochemical Pharmacology, 116218 (2024)
