# Supplementary material for: From biogenesis to deep modeling: a holistic review of miRNA–disease prediction computational methods with experimental comparison
Source: Brief Bioinform. 2026 Jan 19;27(1):bbaf736. doi: 10.1093/bib/bbaf736 (PMC12814990; doi:10.1093/bib/bbaf736)
Supplement: Supplementary_Text_3_bbaf736 [file supplementary_text_3_bbaf736.pdf]

# Supplementary Text 3

## Tracing the origin of miRNA

Figure 1 presents a timeline of key milestones in miRNA research from 1993 to 2025, segmented into five distinct developmental phases. It summarizes the progression from the foundational discoveries in *Caenorhabditis elegans* (*C. elegans*) to the current era of clinical translation and interdisciplinary innovation. Collectively, these advances underscore the critical role of miRNA research in elucidating gene regulation and its profound impact on both biology and medicine.

### Phase I: Discovery and Preliminary Period (1993–2000)

In 1993, the laboratory of Victor Ambros discovered the first miRNA in *C.elegans* [1], a small RNA sequence (`lin-4` miRNA) identified in the `lin-4` gene. At the same time, Ruvkun focused on the regulatory role of the `lin-14` gene in *C.elegans* [2]. Experimental analysis showed that `lin-4` miRNA inhibited the expression of the lin-14 gene by binding to the complementary sequence of the 3' UTR of the lin-14 mRNA, preventing the production of the lin-14 protein. Thus, the role of miRNA in posttranscriptional regulation was established. For seven years after the discovery of `lin-4` miRNA, it was considered an anomaly and was thought to be unique to *C.elegans*. However, in 2000, Ruvkun discovered a second miRNA, the `let-7` miRNA, in *C.elegans*. Subsequent studies confirmed that `let-7` miRNA is conserved in higher animals, including humans [3, 4], highlighting the evolutionary conservation and widespread regulatory importance of miRNAs.

### Phase II: Mechanistic Elucidation and Functional Exploration Period (2001–2006)

In October 2001, research teams led by Thomas Tuschl, David Bartel, and Victor Ambros published seminal studies [5–7], formally coining the term "microRNA" (miRNA) and marking the official launch of miRNA research. This period also witnessed the discovery of `Dicer` as an essential enzyme for miRNA maturation [8, 9]. By 2002, dysregulation of miRNAs in tumor tissues was observed; notably, the deregulation of the `miR-15a/16-1` cluster in chronic lymphocytic leukemia (CLL) underscored their tumor-suppressive functions [10]. This study marked the beginning of a new chapter in the exploration of miRNA-disease associations. In 2003, additional miRNAs were identified in Ruvkun's laboratory [11], followed by the detection of miRNAs in mammalian neurons [12]. Bartel *et al.* further demonstrated the ubiquity of miRNAs in multicellular organisms and their involvement in diverse biological processes, setting the stage for their application as diagnostic and prognostic biomarkers [13, 14]. In 2005, He *et al.* demonstrated that aberrant miRNA expression can directly contribute to tumor formation and growth in vivo [15]. The first study examining cardiac miRNAs emerged in 2006, linking their dysregulation to cardiac remodeling in both mice and humans [16]. This phase was punctuated by the awarding of the Nobel Prize in Physiology or Medicine to Andrew Fire and Craig Mello for their discovery of RNA interference (RNAi), which shares key mechanistic similarities with miRNA-mediated regulation.

### Phase III: Systematic Research and Clinical Exploration Period (2007–2012)

During this phase, miRNA research rapidly advanced in both scope and depth. In 2007, Ruvkun's laboratory identified protein cofactors that interact with miRNAs to exert their gene regulatory functions [17]. Concurrently, aberrant miRNA expression was linked to a variety of neurodegenerative conditions [18–20] as well as autoimmune diseases [21, 22]. In 2008, Victor Ambros and Gary Ruvkun were jointly awarded the Lasker Award for Basic Medical Research in recognition of their pioneering contributions to the field. In 2009, the first proof-of-concept for miRNA delivery as a therapeutic strategy for cancer was demonstrated [23]. Starting in 2010, research shifted toward a deeper mechanistic understanding and broader clinical applications. Studies revealed that miRNAs predominantly mediate mRNA destabilization [24] and that the overexpression of a single miRNA can induce oncogenesis [25]. The discovery of circulating miRNAs in 12 different human body fluids further underscored their potential as non-invasive biomarkers for early disease detection [26].

### Phase IV: Technological Innovations, and Clinical Translation (2013–2024)

Technological innovations, including high-throughput sequencing [27, 28], lateral flow chromatography [29, 30], and Next Generation Sequencing (NGS) [31–33] methods, have since revolutionized the identification and profiling of miRNAs under both physiological and pathological conditions. These advances have enabled detailed investigations into the roles of miRNAs in neurodegenerative disorders [34–36], cardiovascular diseases [37, 38], metabolic syndromes [39, 40], and various cancers [41–43]. With the development and integration of bioinformatics and computer technology, the performance of computational methods for predicting MDAs has been enhanced [44–46]. In a landmark achievement, 2024 witnessed Victor Ambros and Gary Ruvkun being awarded the Nobel Prize in Physiology or Medicine for their seminal discoveries regarding miRNAs and post-transcriptional gene regulation, thereby reaffirming the pivotal role of miRNA research in modern biology.

### Phase V: Multidisciplinary Integration and Future Prospects (2025 and Beyond)

Looking forward, the field is poised for transformative growth through its integration with disciplines such as artificial intelligence, nanotechnology [47], and precision medicine. Emerging research is focusing on the use of targeted gene editing and miRNA-based therapeutics for innovative treatments [48–50] , including potential strategies for restoring hearing function [51–53] and addressing other complex diseases [54, 55]. This multidisciplinary convergence promises to further expand the utility of miRNAs in disease diagnosis, therapy, and prevention, paving the way for the next wave of breakthroughs in biomedical research [56, 57].

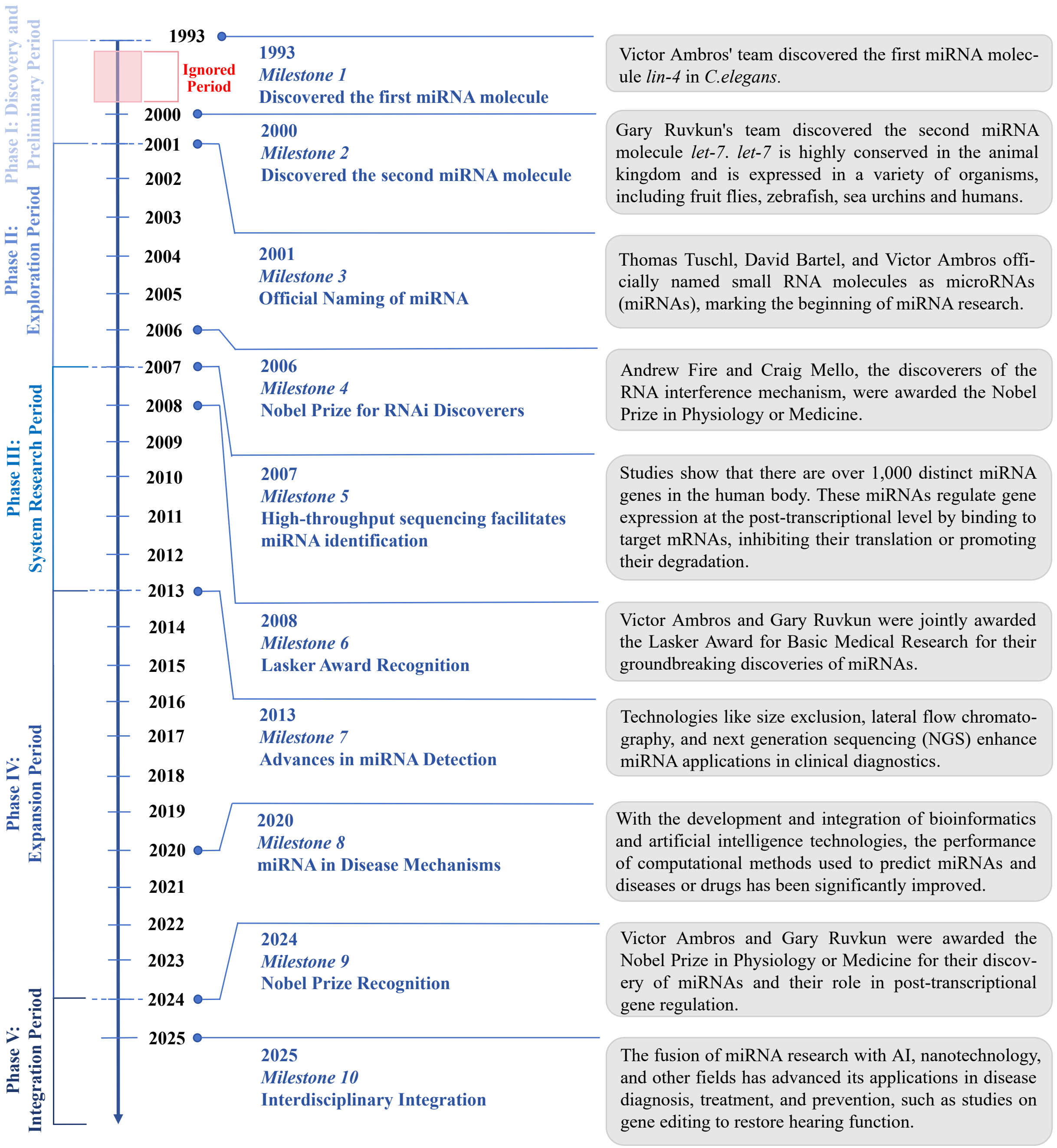


**Fig. 1**: The history of miRNA research: key developments and advancements in miRNA studies, breakthroughs, and emerging directions.

## References


[1] Lee, R.C., Feinbaum, R.L., Ambros, V.: The c. elegans heterochronic gene lin-4 encodes small rnas with antisense complementarity to lin-14. cell **75**(5), 843–854 (1993)

[2] Wightman, B., Ha, I., Ruvkun, G.: Posttranscriptional regulation of the heterochronic gene lin-14 by lin-4 mediates temporal pattern formation in c. elegans. Cell **75**(5), 855–862 (1993)

[3] Reinhart, B.J., Slack, F.J., Basson, M., Pasquinelli, A.E., Bettinger, J.C., Rougvie, A.E., Horvitz, H.R., Ruvkun, G.: The 21-nucleotide let-7 rna regulates developmental timing in caenorhabditis elegans. nature **403**(6772), 901–906 (2000)

[4] Slack, F.J., Basson, M., Liu, Z., Ambros, V., Horvitz, H.R., Ruvkun, G.: The lin-41 rbcc gene acts in the c. elegans heterochronic pathway between the let-7 regulatory rna and the lin-29 transcription factor. Molecular cell **5**(4), 659–669 (2000)

[5] Ambros, V.: Dicing up rnas. Science **293**(5531), 811–813 (2001)

[6] Lee, R.C., Ambros, V.: An extensive class of small rnas in caenorhabditis elegans. science **294**(5543), 862–864 (2001)

[7] Lau, N.C., Lim, L.P., Weinstein, E.G., Bartel, D.P.: An abundant class of tiny rnas with probable regulatory roles in caenorhabditis elegans. Science **294**(5543), 858–862 (2001)

[8] Grishok, A., Pasquinelli, A.E., Conte, D., Li, N., Parrish, S., Ha, I., Baillie, D.L., Fire, A., Ruvkun, G., Mello, C.C.: Genes and mechanisms related to rna interference regulate expression of the small temporal rnas that control c. elegans developmental timing. Cell **106**(1), 23–34 (2001)

[9] Hutvagner, G., McLachlan, J., Pasquinelli, A.E., Bálint, É., Tuschl, T., Zamore, P.D.: A cellular function for the rna-interference enzyme dicer in the maturation of the let-7 small temporal rna. Science **293**(5531), 834–838 (2001)

[10] Calin, G.A., Dumitru, C.D., Shimizu, M., Bichi, R., Zupo, S., Noch, E., Aldler, H., Rattan, S., Keating, M., Rai, K., *et al.*: Frequent deletions and down-regulation of micro-rna genes mir15 and mir16 at 13q14 in chronic lymphocytic leukemia. Proceedings of the national academy of sciences **99**(24), 15524–15529 (2002)

[11] Grad, Y., Aach, J., Hayes, G.D., Reinhart, B.J., Church, G.M., Ruvkun, G., Kim, J.: Computational and experimental identification of c. elegans micrornas. Molecular cell **11**(5), 1253–1263 (2003)

[12] Kim, J., Krichevsky, A., Grad, Y., Hayes, G.D., Kosik, K.S., Church, G.M., Ruvkun, G.: Identification of many micrornas that copurify with polyribosomes in mammalian neurons. Proceedings of the National Academy of Sciences **101**(1), 360–365 (2004)

[13] Bartel, D.P.: Micrornas: genomics, biogenesis, mechanism, and function. cell **116**(2), 281–297 (2004)

[14] Takamizawa, J., Konishi, H., Yanagisawa, K., Tomida, S., Osada, H., Endoh, H., Harano, T., Yatabe, Y., Nagino, M., Nimura, Y., *et al.*: Reduced expression of the let-7 micrornas in human lung cancers in association with shortened postoperative survival. Cancer research **64**(11), 3753–3756 (2004)

[15] He, L., Thomson, J.M., Hemann, M.T., Hernando-Monge, E., Mu, D., Goodson, S., Powers, S., Cordon-Cardo, C., Lowe, S.W., Hannon, G.J., *et al.*: A microrna polycistron as a potential human oncogene. nature **435**(7043), 828–833 (2005)

[16] Van Rooij, E., Sutherland, L.B., Liu, N., Williams, A.H., McAnally, J., Gerard, R.D., Richardson, J.A., Olson, E.N.: A signature pattern of stress-responsive micrornas that can evoke cardiac hypertrophy and heart failure. Proceedings of the National Academy of Sciences **103**(48), 18255–18260 (2006)

[17] Parry, D.H., Xu, J., Ruvkun, G.: A whole-genome rnai screen for c. elegans mirna pathway genes. Current Biology **17**(23), 2013–2022 (2007)

[18] Schaefer, A., O'Carroll, D., Tan, C.L., Hillman, D., Sugimori, M., Llinas, R., Greengard, P.: Cerebellar neurodegeneration in the absence of micrornas. The Journal of experimental medicine **204**(7), 1553–1558 (2007)

[19] Lukiw, W.J.: Micro-rna speciation in fetal, adult and alzheimer's disease hippocampus. Neuroreport **18**(3), 297–300 (2007)

[20] Kim, J., Inoue, K., Ishii, J., Vanti, W.B., Voronov, S.V., Murchison, E., Hannon, G., Abeliovich, A.: A microrna feedback circuit in midbrain dopamine neurons. Science **317**(5842), 1220–1224 (2007)

[21] Sonkoly, E., Wei, T., Janson, P.C., Sääf, A., Lundeberg, L., Tengvall-Linder, M., Norstedt, G., Alenius, H., Homey, B., Scheynius, A., *et al.*: Micrornas: novel regulators involved in the pathogenesis of psoriasis? PloS one **2**(7), 610 (2007)

[22] Dai, Y., Huang, Y.-S., Tang, M., Lv, T.-Y., Hu, C.-X., Tan, Y.-H., Xu, Z.-M., Yin, Y.-B.: Microarray analysis of microrna expression in peripheral blood cells of systemic lupus erythematosus patients. Lupus **16**(12), 939–946 (2007)

[23] Kota, J., Chivukula, R.R., O'Donnell, K.A., Wentzel, E.A., Montgomery, C.L., Hwang, H.-W., Chang, T.-C., Vivekanandan, P., Torbenson, M., Clark, K.R., *et al.*: Therapeutic microrna delivery suppresses tumorigenesis in a murine liver cancer model. Cell **137**(6), 1005–1017 (2009)

[24] Guo, H., Ingolia, N.T., Weissman, J.S., Bartel, D.P.: Mammalian micrornas predominantly act to decrease target mrna levels. Nature **466**(7308), 835–840 (2010)

[25] Medina, P.P., Nolde, M., Slack, F.J.: Oncomir addiction in an in vivo model of microrna-21-induced pre-b-cell lymphoma. Nature **467**(7311), 86–90 (2010)

[26] Weber, J.A., Baxter, D.H., Zhang, S., Huang, D.Y., How Huang, K., Jen Lee, M., Galas, D.J., Wang, K.: The microrna spectrum in 12 body fluids. Clinical chemistry **56**(11), 1733–1741 (2010)

[27] Zhu, E., Zhao, F., Xu, G., Hou, H., Zhou, L., Li, X., Sun, Z., Wu, J.: mirtools: microrna profiling and discovery based on high-throughput sequencing. Nucleic acids research **38**(suppl_2), 392–397 (2010)

[28] Chien, C.-H., Sun, Y.-M., Chang, W.-C., Chiang-Hsieh, P.-Y., Lee, T.-Y., Tsai, W.-C., Horng, J.-T., Tsou, A.-P., Huang, H.-D.: Identifying transcriptional start sites of human micrornas based on high-throughput sequencing data. Nucleic acids research **39**(21), 9345–9356 (2011)

[29] Zheng, W., Yao, L., Teng, J., Yan, C., Qin, P., Liu, G., Chen, W.: Lateral flow test for visual detection of multiple micrornas. Sensors and Actuators B: Chemical **264**, 320–326 (2018)

[30] Chen, M., Ma, E., Xing, Y., Xu, H., Chen, L., Wang, Y., Zhang, Y., Li, J., Wang, H., Zheng, S.: Dual-modal lateral flow test strip assisted by near-infrared-powered nanomotors for direct quantitative detection of circulating microrna biomarkers from serum. ACS sensors **8**(2), 757–766 (2023)

[31] Motameny, S., Wolters, S., Nürnberg, P., Schumacher, B.: Next generation sequencing of mirnas–strategies, resources and methods. Genes **1**(1), 70–84 (2010)

[32] Tam, S., De Borja, R., Tsao, M.-S., McPherson, J.D.: Robust global microrna expression profiling using next-generation sequencing technologies. Laboratory investigation **94**(3), 350–358 (2014)

[33] Galluzzo, A., Gallo, S., Pardini, B., Birolo, G., Fariselli, P., Boretto, P., Vitacolonna, A., Peraldo-Neia, C., Spilinga, M., Volpe, A., *et al.*: Identification of novel circulating micrornas in advanced heart failure by next-generation sequencing. ESC Heart Failure **8**(4), 2907–2919 (2021)

[34] Lau, P., Strooper, B.: Dysregulated micrornas in neurodegenerative disorders. In: Seminars in Cell & Developmental Biology, vol. 21, pp. 768–773 (2010). Elsevier

[35] Junn, E., Mouradian, M.M.: Micrornas in neurodegenerative diseases and their therapeutic potential. Pharmacology & therapeutics **133**(2), 142–150 (2012)

[36] Berg, M., Krauskopf, J., Ramaekers, J., Kleinjans, J., Prickaerts, J., Briedé, J.: Circulating micrornas as potential biomarkers for psychiatric and neurodegenerative disorders. Progress in neurobiology **185**, 101732 (2020)

[37] Small, E.M., Frost, R.J., Olson, E.N.: Micrornas add a new dimension to cardiovascular disease. Circulation **121**(8), 1022–1032 (2010)

[38] Barwari, T., Joshi, A., Mayr, M.: Micrornas in cardiovascular disease. Journal of the American College of Cardiology **68**(23), 2577–2584 (2016)

[39] Schroen, B., Heymans, S.: Small but smart—micrornas in the centre of inflammatory processes during cardiovascular diseases, the metabolic syndrome, and ageing. Cardiovascular research **93**(4), 605–613 (2012)

[40] Rottiers, V., Näär, A.M.: Micrornas in metabolism and metabolic disorders. Nature reviews Molecular cell biology

**13**(4), 239–250 (2012)

[41] Bach, D.-H., Hong, J.-Y., Park, H.J., Lee, S.K.: The role of exosomes and mirnas in drug-resistance of cancer cells. International journal of cancer **141**(2), 220–230 (2017)

[42] Najminejad, H., Kalantar, S.M., Abdollahpour-Alitappeh, M., Karimi, M.H., Seifalian, A.M., Gholipourmalekabadi, M., Sheikhha, M.H.: Emerging roles of exosomal mirnas in breast cancer drug resistance. IUBMB life **71**(11), 1672–1684 (2019)

[43] Alharbi, M., Zuñiga, F., Elfeky, O., Guanzon, D., Lai, A., Rice, G.E., Perrin, L., Hooper, J., Salomon, C.: The potential role of mirnas and exosomes in chemotherapy in ovarian cancer. Endocrine-related cancer **25**(12), 663–685 (2018)

[44] Wen, S., Liu, Y., Yang, G., Chen, W., Wu, H., Zhu, X., Wang, Y.: A method for mirna-disease association prediction using machine learning decoding of multi-layer heterogeneous graph transformer encoded representations. Scientific Reports **14**(1), 20490 (2024)

[45] Zhang, C., Li, Y., Dong, Y., Chen, W., Yu, C.: Prediction of mirna-disease associations based on pca and cascade forest. BMC bioinformatics **25**(1), 386 (2024)

[46] Ouyang, D., Miao, R., Zeng, J., Li, X., Ai, N., Wang, P., Hou, J., Zheng, J.: Splhrnmtf: robust orthogonal non-negative matrix tri-factorization with self-paced learning and dual hypergraph regularization for predicting mirna-disease associations. BMC genomics **25**(1), 885 (2024)

[47] Chai, M., Gao, B., Wang, S., Zhang, L., Pei, X., Yue, B., Zhen, X., Zhang, M.: Leveraging plant-derived nanovesicles for advanced nucleic acid-based gene therapy. Theranostics **15**(1), 324 (2025)

[48] Visscher, P.M., Gyngell, C., Yengo, L., Savulescu, J.: Heritable polygenic editing: the next frontier in genomic medicine? Nature, 1–9 (2025)

[49] Ghassemifard, L., Hasanlu, M., Parsamanesh, N., Atkin, S.L., Almahmeed, W., Sahebkar, A.: Cell therapies and gene therapy for diabetes: current progress. Current Diabetes Reviews **21**(8), 130524229899 (2025)

[50] Ortega, A., Chernicki, B., Ou, G., Parmar, M.S.: From lab bench to hope: Emerging gene therapies in clinical trials for alzheimer's disease. Molecular Neurobiology **62**(1), 1112–1135 (2025)

[51] Lv, X., Yang, C., Li, X., Liu, Y., Yang, Y., Jin, T., Chen, Z., Jia, J., Wang, M., Li, L.: Ferroptosis and hearing loss: from molecular mechanisms to therapeutic interventions. Journal of Enzyme Inhibition and Medicinal Chemistry **40**(1), 2468853 (2025)

[52] Awang, D., Danzeng, K., Wang, T., Deji, Q., Huang, M., Ren, H., Liu, X., Zhao, B., Gongga, L.: Circrna-mirna-mrna regulatory network in high-altitude hypobaric hypoxia-induced hearing impairment and hearing acclimatization. Brazilian Journal of Otorhinolaryngology **91**(2), 101557 (2025)

[53] Gu, X., Jiang, M., Chen, W.: mir-145b/ap2b1 axis contributes to noise-induced sensorineural hearing loss in a male mouse model. Cell Biochemistry and Biophysics, 1–9 (2025)

[54] Du, G., Yao, Y., Chen, W., Lin, Y., He, Y., Zhou, M., Cai, X.: Tetrahedral framework nucleic acid-based delivery system for mirna-124 and stroke-homing peptide: Targeted therapy for acute ischemic stroke. ACS Applied Materials & Interfaces (2025)

[55] Tian, X., Zhou, M., Zhang, J., Huang, X., Jiang, D., Liu, J., Zhang, Q., Chen, D., Hu, Q.: Mechanism of lncrna-mirna in renal intrinsic cells of diabetic kidney disease and potential therapeutic direction. DNA and Cell Biology (2025)

[56] Baker, A.H., Giacca, M., Thum, T.: mirna discovery to therapy: The field is sufficiently mature to assess the value of mirna-based therapeutics. Molecular Therapy **33**(1), 3–4 (2025)

[57] Qian, Y., Liu, C., Zeng, X., Li, L.-C.: Rnaa: Mechanisms, therapeutic potential, and clinical progress. Molecular Therapy Nucleic Acids **36**(2) (2025)
