# Supplementary material for: From biogenesis to deep modeling: a holistic review of miRNA–disease prediction computational methods with experimental comparison
Source: Brief Bioinform. 2026 Jan 19;27(1):bbaf736. doi: 10.1093/bib/bbaf736 (PMC12814990; doi:10.1093/bib/bbaf736)
Supplement: Supplementary_Text_4_bbaf736 [file supplementary_text_4_bbaf736.pdf]

Supplementary Text 4

1 Disease Similarity Matrixes

1.1 Disease Semantic Similarity (DSS)

I. The Ontology-based disease semantic similarity ($\mathbf{DSS}_1$). To compute the semantic similarity between diseases, Tang *et al.* [1] adopted the strategy based on directed acyclic graphs (DAGs) proposed by Wang *et al.* [2]. For any disease d, its DAG is defined as $\mathrm{DAG}(d) = (d, T(d), E(d))$, where $T(d)$ is the set of d and all its ancestor nodes, and $E(d)$ is the set of directed edges. The semantic contribution of a node $t \in T(d)$ to d is then evaluated recursively by:

$$D_d(t) = \begin{cases} 1, & t = d \\ \max\{\Delta \cdot D_d(t') \mid t' \in \text{children of } t\}, & \text{otherwise} \end{cases} \tag{1}$$

where the decay factor $\Delta = 0.5$. Consequently, the pairwise semantic similarity between diseases d_i and d_j is given by:

$$\mathrm{DSS}_1(d_i, d_j) = \frac{\sum\limits_{t \in T(d_i) \cap T(d_j)} \big(D_{d_i}(t) + D_{d_j}(t)\big)}{\sum\limits_{t \in T(d_i)} D_{d_i}(t) + \sum\limits_{t \in T(d_j)} D_{d_j}(t)} \tag{2}$$

The resulting matrix $\mathrm{DSS}_1(d_i, d_j) \in \mathbb{R}^{D \times D}$ constitutes the semantic view of the D diseases.

II. An enhanced semantic similarity $\mathbf{DSS}_2$. However, DSS_1 ignores the differences in the importance of semantic contributions among different diseases, neglecting the fact that rare diseases appearing in fewer DAGs should receive higher weights and thus higher semantic contribution values. According to previous research [3], the second semantic contribution is defined as:

$$D(d_i, d_k) = -\log\left(\frac{\text{the number of DAGs including } d_k}{\text{the number of disease}}\right) \tag{3}$$

Therefore, the second semantic value $\mathrm{SV}(d_i)$ of disease d_i and the second semantic similarity $\mathrm{DSS}_2(d_i, d_j)$ between disease d_i and d_j are:

$$\mathrm{SV}(d_i) = \sum_{d_k \in T(d_i)} D(d_i, d_k) \tag{4}$$

$$\mathrm{DSS}_2(d_i, d_j) = \frac{\sum\limits_{d_t \in T(d_i) \cap T(d_j)} \big(D(d_i, d_t) + D(d_j, d_t)\big)}{\mathrm{SV}(d_i) + \mathrm{SV}(d_j)} \tag{5}$$

1.2 Disease Functional Similarity (DFS)

Similar to miRNA functional similarity calculations based on miRNA-gene association, disease functional similarity is calculated using a shared gene set of diseases. Let $\mathbf{G}_i$ and $\mathbf{G}_j$ denote the genes associated with diseases d_i and d_j, respectively. The target-based similarity between the two diseases d_i and d_j is given by:

$$DFS(d_i, d_j) = \frac{\sum_{g \in \mathbf{G}_i} S(g, \mathbf{G}_j) + \sum_{g \in \mathbf{G}_j} S(g, \mathbf{G}_i)}{|\mathbf{G}_i| + |\mathbf{G}_j|} \tag{6}$$

$$S(g, \mathbf{G}) = \max_{g' \in \mathbf{G}} s(g, g') \tag{7}$$

where $\mathbf{G}_i$ and $\mathbf{G}_j$ are the sets of genes associated with d_i and d_j, respectively. $s(g, g')$ is the gene-gene similarity score. The resulting matrix $DFS \in \mathbb{R}^{N \times N}$ represents the functional similarity of N diseases.

2 miRNA Similarity Matrixes

2.1 miRNA Functional Similarity (MFS)

miRNA Functional Similarity (MFS) matrix is based on the assumption that miRNAs associated with similar diseases have similar effects. There are three ways to calculate the MFS matrix:

I. based on known MDAs [4]. To calculate miRNA functional similarity, Li *et al.* [4] employs a disease-semantic-structure-based approach that incorporates the directionality of miRNA regulation. First, semantic similarities among diseases are derived from their MeSH-directed acyclic graphs (DAGs); each miRNA is then represented by a signed

semantic feature vector that distinguishes up-regulation ($r = 0$) from down-regulation ($r = 1$). These vectors are subsequently augmented with cross-disease-set semantic similarities, and the final functional similarity is evaluated by cosine similarity. The key equations are:

$$f_{m_i} = \{(-1)^r DV(d_{i1}), \ldots, (-1)^r DV(d_{in})\} \tag{8}$$

$$\mathrm{COS}(m_i, m_j) = \frac{f'_{m_i} \cdot f'_{m_j}{}^{\top}}{\|f'_{m_i}\| \|f'_{m_j}\|} \tag{9}$$

where $DV(d) = \sum_{t \in T_d} D_d(t)$ denotes the semantic value of disease d. $D_d(t)$ is the semantic contribution of associated disease t to disease d with decay factor $\Delta = 0.5$. T_d represents the set of all ancestor nodes of d, including disease d itself. f'_{m_i} and f'_{m_j} are the semantic similarities between any two diseases associated with m_i and m_j, respectively, serving as the enhanced feature vector for calculating the final miRNA functional similarity. For the specific calculation process of the enhanced feature vector, please refer to the paper [4].

II. based on disease functional similarity [5]. Based on the assumption that miRNAs associated with similar diseases may have similar functions, the miRNA functional similarity score can be calculated according to disease semantic similarity [4]. Then, we can build a miRNA functional similarity matrix MFS. $MFS(m_i, m_j)$ denotes each element in the matrix MFS, which also represents the miRNA functional similarity score between miRNAs m_i and m_j. Finally, MFS can be calculated by the following formula:

$$MFS(m_i, m_j) = \frac{\sum_{d \in D(m_i)} DSS(d, d_j^*) + \sum_{d \in D(m_j)} DSS(d, d_i^*)}{|D(m_i)| + |D(m_j)|} \tag{10}$$

$$d_i^* = \max_{d_i \in D(m_i)} DSS(d, d_i) \tag{11}$$

where $D(m_i)$ denotes the set of diseases that are associated with m_i, and $|D(m_i)|$ represents the number of elements in the set $D(m_i)$.

III. based on miRNA-gene association [1, 6]. Previous studies [1] have used miRNA-gene associations to calculate the functional similarity of miRNAs. The gene functional interaction network is obtained from HumanNet [1], which provides log-likelihood scores (LLS) reflecting the probability of a functional linkage between genes. We first compute the normalised gene-gene similarity, denoted as LLS_n, by performing min-max scaling on the original LLS values. The gene similarity graph is subsequently defined as:

$$S(g_i, g_j) = \begin{cases} 1, & g_i = g_j \\ LLS_n(g_i, g_j), & e(g_i, g_j) \in \mathcal{S}_{\mathrm{HumanNet}} \\ 0, & \text{otherwise} \end{cases} \tag{12}$$

where $\mathcal{S}_{\mathrm{HumanNet}}$ is the set of all HumanNet edges and $e(g_i, g_j)$ denotes the edge between genes g_i and g_j. Next, the similarity between a gene g_s and a gene set $\mathbf{G}_S = \{g_{s1}, g_{s2}, \ldots, g_{sk}\}$ is defined as:

$$S(g_s, \mathbf{G}_S) = \max_{1 \le i \le k} S(g_s, g_{si}) \tag{13}$$

The functional similarity between two miRNAs m_i and m_j is then calculated by:

$$G^{\mathrm{m}}(m_i, m_j) = \frac{\sum_{g \in \mathbf{G}_i} S(g, \mathbf{G}_j) + \sum_{g \in \mathbf{G}_j} S(g, \mathbf{G}_i)}{|\mathbf{G}_i| + |\mathbf{G}_j|} \tag{14}$$

where $\mathbf{G}_i$ and $\mathbf{G}_j$ correspond to the gene sets linked to miRNAs m_i and m_j, respectively. The resulting matrix $\mathbf{G}^{\mathrm{m}}$ forms the miRNA functional similarity view.

2.2 miRNA Sequence Similarity (MSS)

I. based on the Needleman-Wunsch algorithm. To construct miRNA sequence-level similarity, the Needleman-Wunsch algorithm is first used to align the seed regions of all miRNAs [1]. The raw alignment scores are assembled into a matrix $\mathbf{M}^s$. To confine the values to a global $[0, 1]$ scale, we perform min-max normalisation:

$$\widetilde{\mathbf{M}}^s(m_i, m_j) = \frac{\mathbf{M}^s(m_i, m_j) - \mathbf{M}^s_{\min}}{\mathbf{M}^s_{\max} - \mathbf{M}^s_{\min}} \tag{15}$$

where $\mathbf{M}^s_{\min}$ and $\mathbf{M}^s_{\max}$ denote the smallest and largest entries in $\mathbf{M}^s$, respectively. The final sequence similarity is then defined as:

$$\mathbf{G}^s_m(m_i, m_j) = \begin{cases} 1, & m_i = m_j \\ \widetilde{\mathbf{M}}^s(m_i, m_j), & m_i \neq m_j \end{cases} \tag{16}$$

The matrix $\mathbf{G}^s_m \in \mathbb{R}^{M \times M}$ serves as the sequence-based view of the miRNA set, with M being the total number of miRNAs.

II. based on the Smith Waterman algorithm. Mature miRNA sequences were retrieved from miRBase [7]. The `pairwiseAlignment` function from the R language's *Biostrings* package was used to calculate a similarity score for each pair of complete mature miRNA sequences, specifically a base similarity score. The following settings were applied when processing the mature miRNA sequences:

- gapOpening penalty = 5,
- gapExtension penalty = 2,
- match score = 1,
- mismatch score = −1.

The alignment score in the `pairwiseAlignment` function is computed via dynamic programming. For global alignment, the Needleman-Wunsch algorithm is employed, whose recurrence is

$$F(i,j) = \max \begin{cases} F(i-1, j-1) + \mathrm{s}(A_i, B_j), & \text{(match/mismatch)} \\ F(i-1, j) + \text{gapOpening}, & \text{(gap in sequence miRNA } B) \\ F(i, j-1) + \text{gapExtension}, & \text{(gap in sequence miRNA } A) \end{cases} \tag{17}$$

$$s(A_i, B_j) = \begin{cases} +1, & i = j \\ -1, & i \neq j \end{cases} \tag{18}$$

where $F(i,j)$ is the optimal score up to position i in sequence miRNA A and position j in sequence miRNA B. Finally, the raw alignment scores for each miRNA pair (i,j) was normalized to $[0,1]$ by:

$$\text{Score}(i,j) = \frac{\text{Score}(i,j) - \text{Score}_{\min}}{\text{Score}_{\max} - \text{Score}_{\min}} \tag{19}$$

where $\text{Score}_{\min}$ and $\text{Score}_{\max}$ are the minimum and maximum scores among all pairs. Combining the similarity scores of all miRNA pairs yields a sequence similarity matrix.

3 Gaussian Interaction Profile Kernel for miRNAs and Diseases

For a given miRNA m_i, a binary vector $IP(m_i)$ was extracted from the known MDAs to represent associations between miRNA m_i and each disease. Then, the miRNAs GIPK similarity $MGIPK(m_i, m_j)$ between miRNAs m_i and m_j can be presented as follows:

$$MGIPK(m_i, m_j) = \exp\left(-\gamma_m \|IP(m_i) - IP(m_j)\|^2\right) \tag{20}$$

where the parameter γ_m controls the kernel bandwidth, which can be calculated using the following equation:

$$\gamma_m = \frac{\gamma'_m}{\frac{1}{M}\sum_{i=1}^{M}\|IP(m_i)\|^2} \tag{21}$$

where M represents the number of miRNAs. Here, γ'_m is set to 1 according to the previous work [8]. Similarly, the diseases GIPK similarity $DGIPK(d_i, d_j)$ between diseases d_i and d_j can be calculated based on the following two equations:

$$DGIPK(d_i, d_j) = \exp\left(-\gamma_d \|IP(d_i) - IP(d_j)\|^2\right) \tag{22}$$

$$\gamma_d = \frac{\gamma'_d}{\frac{1}{D}\sum_{j=1}^{D}\|IP(d_j)\|^2} \tag{23}$$

where D denotes the number of diseases and $\gamma'_d = 1$.

References

[1] Tang, X., Luo, J., Shen, C., Lai, Z.: Multi-view multichannel attention graph convolutional network for mirna–disease association prediction. Briefings in Bioinformatics **22**(6), 174 (2021)

[2] Wang, D., Wang, J., Lu, M., Song, F., Cui, Q.: Inferring the human microrna functional similarity and functional network based on microrna-associated diseases. Bioinformatics **26**(13), 1644–1650 (2010)

[3] Xuan, P., Han, K., Guo, M., Guo, Y., Li, J., Ding, J., Liu, Y., Dai, Q., Li, J., Teng, Z., *et al.*: Prediction of micrornas associated with human diseases based on weighted k most similar neighbors. PloS one **8**(8), 70204 (2013)

[4] Li, J., Zhang, S., Wan, Y., Zhao, Y., Shi, J., Zhou, Y., Cui, Q.: Misim v2. 0: a web server for inferring microrna functional similarity based on microrna-disease associations. Nucleic acids research **47**(W1), 536–541 (2019)

[5] Ouyang, D., Liang, Y., Wang, J., Li, L., Ai, N., Feng, J., Lu, S., Liao, S., Liu, X., Xie, S.: Hgclamir: Hypergraph contrastive learning with attention mechanism and integrated multi-view representation for predicting mirna-disease associations. PLOS Computational Biology **20**(4), 1011927 (2024)

[6] Xiao, Q., Luo, J., Liang, C., Cai, J., Ding, P.: A graph regularized non-negative matrix factorization method for identifying microrna-disease associations. Bioinformatics **34**(2), 239–248 (2018)

[7] Kozomara, A., Griffiths-Jones, S.: mirbase: annotating high confidence micrornas using deep sequencing data. Nucleic acids research **42**(D1), 68–73 (2014)

[8] Zhao, Y., Chen, X., Yin, J.: Adaptive boosting-based computational model for predicting potential mirna-disease associations. Bioinformatics **35**(22), 4730–4738 (2019)
